# Mao’s Cultural War: The Origins of the Cultural Revolution Revisited

**DOI:** 10.1177/00977004261431551

**Published:** 2026-05-19

**Authors:** Yiching Wu

**Affiliations:** 1 Department of East Asian Studies, University of Toronto, Toronto, ON, Canada

**Keywords:** Cultural Revolution, intellectuals, early Cultural Revolution, Mao Zedong, May 16 Notice, Sixteen Articles

## Abstract

The Great Proletarian Cultural Revolution was a sweeping mass assault that shook the political foundations of China’s Leninist party-state. But why was it called a “cultural revolution”? What, precisely, was “cultural” about Mao’s last revolutionary endeavor? Focusing on the ideological and cultural struggles that preceded and shaped its early phase, this article challenges the prevailing narrative that assumes Mao conceived of the Cultural Revolution as an insurgent political movement from the outset. Instead, it contends that the movement initially took form as a radical “cultural revolution” in the literal sense—rooted in Mao’s longstanding vision of ideological and cultural transformation. What began as a targeted intervention within the cultural domain, however, escalated into a far-reaching political upheaval and systemic crisis, ultimately exceeding Mao’s original expectations. By reconstructing Mao’s intent in historical context and treating “cultural revolution” as a distinct historiographical category, this article offers a more historically grounded account of the Cultural Revolution’s origins and early dynamics.

The Cultural Revolution was an unparalleled political crisis that destabilized the foundations of the People’s Republic of China (PRC). Mao Zedong, asserting that “class enemies” had infiltrated the Chinese Communist Party (CCP), called for their destruction through a revolutionary mass movement. This resulted in the near-total paralysis of the party-state, as mass insurgencies from below, compounded by elite dysfunction at the top, plunged the country into an extraordinary crisis. Yet what made the Cultural Revolution unique was that this crisis originated at the apex of the state itself: Mao himself questioned the party’s legitimacy, unleashed destructive social forces, and provided the ideological vocabulary for challenging its authority. As Roderick MacFarquhar memorably puts it, “Never before had a dictator unleashed the forces of society against the state which he himself had created. This was indeed the mother of all mass movements” (MacFarquhar, 1997: 465).

Among the many questions that have long puzzled China scholars, none is more vexing than this: why and how did Mao, in the final decade of his rule, come to unleash a vast upheaval that nearly destroyed what he had done so much to create? MacFarquhar’s *The Origins of the Cultural Revolution* remains the most authoritative account, tracing the escalating divisions within the CCP leadership from the late 1950s to the mid-1960s. However, since the appearance of the last volume of his trilogy nearly three decades ago—and particularly following *Mao’s Last Revolution*, his collaboration with Michael Schoenhals, twenty years ago (MacFarquhar and Schoenhals, 2006)—a wealth of new evidence has emerged, offering opportunities to refine and expand our understanding of the period.

Existing scholarship on the origins of the Cultural Revolution generally falls into two divergent but at times overlapping strands. The first one focuses on CCP leadership conflicts that intensified in the aftermath of the Great Leap Forward. In this view, the Cultural Revolution was a Machiavellian maneuver by Mao to eliminate his alleged rivals and reclaim lost authority, which he achieved by directly mobilizing the masses to circumvent the bureaucracy (MacFarquhar, 1987, 1997; Panstov and Levine, 2012; Dikötter, 2016). In contrast, the other approach foregrounds ideology over personal power. In this view, the Cultural Revolution was Mao’s attempt to reinvigorate revolutionary fervor by unleashing mass mobilization against what he perceived as an ossified and increasingly self-protective bureaucratic apparatus. This interpretation highlights Mao’s view that the party had succumbed to organizational routinization, displacing the populist charismatic authority that had once animated the revolution. Though ultimately futile, this endeavor reflected Mao’s enduring belief that renewed ideological commitment and popular activism could counter the tendencies of bureaucratic institutionalization and revive the revolution’s transformative vitality (Dittmer, 1987; Harding, 1981; Kraus, 1981; Lee, 1978; Schram, 1989; Schurmann, 1968; Tsou, 1986).

Despite considerable divergences, scholarly accounts of the Cultural Revolution’s origins and early development have, for the most part, coalesced around a durable historiographical consensus structured by several interlocking premises. First, these narratives attribute the Cultural Revolution either to factional struggles within the CCP leadership or to Mao’s ideological radicalism. Second, they depict Mao’s ideological vision as coherent, consistent, and largely systematic, rather than contingent or situational.^1^ Third, they portray Mao as a master strategist who deliberately orchestrated the movement to purge his rivals, whether real or imagined. Finally, they reconstruct the movement’s early trajectory as a carefully staged, phased, and linear process—beginning with cultural and ideological struggles, then expanding into mass movements, and culminating in widespread political upheaval.

This narrative and explanatory paradigm remains dominant in Cultural Revolution historiography. In the last volume of his seminal *The Origins of the Cultural Revolution*, MacFarquhar argues that as early as late 1962, “one can detect elements of the thinking which would lead eventually to his [Mao’s] decision to launch the Cultural Revolution” (MacFarquhar, 1997: 277). By late 1964 and early 1965, MacFarquhar contends, Mao “evidently felt that the combination of the ideological threat to China and the political threat to himself now demanded decisive action” (MacFarquhar, 1997: 432). By this point, he suggests, it had become clear that Mao had already envisioned a movement far more consequential than the disciplining of minor bureaucratic deviations—one directed instead against high-level “capitalist roaders” within the party. Yet, he maintains, Mao—“too experienced a guerrilla fighter to tip his hand”—proceeded with strategic patience, requiring nearly twenty months, from January 1965 to August 1966, to methodically bring this objective to fruition (MacFarquhar and Schoenhals, 2006: 13).

MacFarquhar famously casts the Cultural Revolution’s emergence as a grand political drama, with Mao as both playwright and director. Within this dramaturgical framework, the movement’s origins and early formation are arranged as a prologue followed by a succession of acts and scenes: the prologue unfolds with the opening salvo on *Hai Rui Dismissed from Office* (November 1965), followed by Act I, which includes a series of pivotal scenes—the instigation of the iconic “first big-character poster” at Peking University (late May 1966), Mao’s emblematic swim in the Yangtze (mid-July), the dismissal of Liu Shaoqi’s work teams (late July), and, culminating in Mao’s “bombarding the headquarters,” the dramatic confrontations at the CCP’s Eleventh Plenum (early August). Act II commenced with the spectacular rise of the Red Guards (mid- to late August) (MacFarquhar, 1997: 461–62). This paradigm has long shaped the field, presenting the early Cultural Revolution as a deliberately scripted drama that foregrounds Mao’s foresight and a tightly sequenced, teleological unfolding. Yet, as this article argues, the continued dominance of this framework warrants critical reassessment and invites alternative approaches that extend beyond its methodological constraints.

## Why “Cultural” Revolution?

This article argues that prevailing narratives must grapple with a critical question that resists easy resolution: Why—and how—did a mass political mobilization aimed at inciting rebellion against the party-state emerge from conflicts rooted in the cultural and ideological domains? Why was this upheaval cast as “cultural,” and what, after all, was “cultural” about the Cultural Revolution?

Evidence suggests that even for Mao, the term “Cultural Revolution” was not self-evident. In a conversation with Albanian visitors in December 1968, he downplayed the term’s specificity, remarking that “Cultural Revolution was merely a general label; in essence, the movement was a political and economic revolution.” This semantic looseness persisted. At the CCP’s Ninth National Congress in April 1969—where the Cultural Revolution was officially declared a “great victory”—Mao offered a notably perfunctory, even circular, justification: “Calling it the Cultural Revolution may be appropriate because the movement began with a cultural revolution” (Documentation Research Office of the Central Committee of the Chinese Communist Party, 2013: 6.146, 242).

This conceptual and semantic ambiguity raises the central question: Was the Cultural Revolution initially conceived as a distinct revolutionary phenomenon, or did it in the end exceed its original framing and scope? Notably, this uncertainty was not confined to Mao. Some student rebels likewise questioned the label, arguing that it failed to accurately characterize the movement. As Yang Xiaokai, an eighteen-year-old militant student from Hunan, remarked in 1967, “This revolution definitely can’t be called a ‘cultural revolution,’ but for the present time, we have no other term but to refer to it as such” (quoted in Wu, 2014: 174).

This article revisits a largely overlooked yet fundamental question: Why was a sweeping political assault on the party-state called a “cultural” revolution in the first place? How did its architects and participants, at the movement’s outset, understand its meaning and significance? I argue that this is not merely a terminological question but signals deeper interpretive and methodological problems that call for re-examining longstanding assumptions about the Cultural Revolution’s nature, scope, and early dynamics.^2^

Scholars have long recognized that the Cultural Revolution opened with confrontations between Maoist radicals and China’s cultural-intellectual establishment, including the party’s propaganda and ideological apparatus. These early skirmishes were deeply intertwined with the radical cultural currents of the preceding years (Goldman, 1966, 1973; Dikötter, 2016: 27–41). Likewise, ambitious efforts at cultural transformation have been widely acknowledged as central to the Cultural Revolution’s broader agendas. Wang Zheng, for instance, highlights this perspective in her important study of feminist socialist culture in the PRC, notably titling one chapter “The Cultural Origins of the Cultural Revolution” (Wang, 2017: 199–220). Similarly, Patricia Thornton characterizes the pre-1966 radical cultural currents as a “cultural revolution before the Cultural Revolution” (Thornton, 2019: 55). Both Paul Clark and Barbara Mittler further reinforce this perspective by tracing the long pre-1966 trajectory of cultural radicalism, showing how various post-1966 efforts to reshape cultural production extended an earlier arc of cultural experimentation. Benjamin Kindler has recently deepened this line of inquiry by reconstructing the genealogy of Maoist cultural politics from the Yan’an period through the 1970s, demonstrating that the project of radical cultural transformation—centered on artistic production and intellectual subjectivity—was a sustained ideological undertaking within Chinese socialism (Kindler, 2025). Taken together, these accounts underscore that Cultural Revolution culture built on, rather than inaugurated, the party’s longstanding attempts to remake the cultural sphere (Clark, 2008; Mittler, 2013).

Yet, despite these important contributions, existing scholarship has not fully accounted for how such projects of radical cultural transformation came to catalyze mass rebellions that later turned against party bureaucracies and officials. Even when culture is recognized as formative, the analytical passage between cultural radicalism and political upheaval remains largely underdeveloped. All too often, the “culture” of the Cultural Revolution has been treated as analytically separable from the political conflicts that defined the era, sometimes quite deliberately so. Mittler, who has forcefully argued for taking culture seriously as a field of inquiry, has significantly advanced our understanding of Cultural Revolution–era culture and art. However, she states that she will “avoid politics” because MacFarquhar and Schoenhals have already treated that terrain extensively (Mittler, 2013: 6). This form of historiographical compartmentalization—treating culture and politics as discrete domains and reinforced by a division of labor between cultural and political historians—risks obscuring a more fundamental dynamic: Cultural radicalism and political upheaval were not merely parallel phenomena but mutually implicated, each shaping and, at times, unsettling the other.

Most historical studies maintain that the Cultural Revolution’s cultural and political dimensions were inextricably linked, with Mao strategically weaponizing cultural debates to advance his political agenda. The late 1965 controversy over Yao Wenyuan’s denunciation of *Hai Rui Dismissed from Office*, a Peking opera written by historian Wu Han, is widely regarded as the movement’s opening salvo. For many, this episode exemplifies Mao’s calculated move of using cultural controversy to undermine the party leadership and attack political rivals. Mao, in the words of MacFarquhar, “was only biding his time for a final assault” on the party’s central leadership (MacFarquhar, 1997: 456). In this reading, the cultural wrangling of late 1965 and early 1966 appears as a choreographed prelude to a “final assault,” a sequence whose coherence is discernible only in retrospect. Yet while this interpretation highlights important factional dynamics, its presumption of Mao’s strategic intent rests more on conjecture than on demonstrable evidence and remains difficult, if not impossible, to substantiate.

While mainstream historiography tends to portray the Cultural Revolution’s early cultural thrusts in instrumental, political terms, an alternative body of scholarship has sought to reinterpret Mao’s objective through a different lens. Alessandro Russo, for instance, contends that Mao’s initial cultural offensive was not simply an instrument of factional maneuvering but a deliberate effort to emancipate mass politics from the ideological authority of the socialist bureaucracy by undermining its “central cultural apparatus.” In this view, it was precisely within the state cultural apparatus, such as education, journalism, and publishing, in Russo’s words, that Mao confronted “the strongest, and the most equivocal, opposition to what he considered crucial political issues” (Russo, 2020: 104–105). Russo’s view resonates, albeit in a different register, with Joel Andreas’s account of the Cultural Revolution as a struggle over the institutional foundations of cultural and political power. Andreas emphasizes that universities and schools had become key sites for the accumulation and reproduction of cultural capital, thereby reinforcing emerging hierarchies and the formation of what he terms the “red and expert elite” within China’s socialist order (Andreas, 2009: 12). From this perspective, the Cultural Revolution’s early assault on the educational system appears as an effort to disrupt the cultural mechanisms through which privilege and authority were consolidated. Taken together, these interpretations shift attention from factional rivalry and toward the cultural stakes of the Cultural Revolution. Yet in treating the cultural campaign as the expression of Mao’s preconceived political vision, they risk attributing to the movement a unity of purpose and ideological consistency that the historical evidence does not sustain.

Maurice Meisner was another scholar who treated Mao’s cultural concerns seriously and, importantly, on their own terms. His attention to what he dubbed “cultural revolution,” which he situated within China’s *longue durée* twentieth-century trajectory, opened an interpretive avenue that few scholars of his generation pursued: that Mao’s bid to reorder the cultural and ideological spheres in 1965–1966 might be understood as an episode of radical cultural activism with its own logic and objectives (Meisner, 1999: 295–300). Yet Meisner ultimately folded this insight back into a continuous political narrative. For him, the Cultural Revolution stood as the culmination of Mao’s protracted struggle against what he called a “Thermidorean” drift following the failure of the Great Leap Forward. Even as he acknowledged that the movement began as a cultural initiative, Meisner reabsorbed that beginning into a unified account in which the movement quickly turned “explicitly political” (Meisner, 1999: 314). In this sense, the interpretive opening that Meisner briefly glimpsed—the possibility of disentangling the cultural project from the subsequent political explosion—was prematurely closed, as his synthesis reconsolidated culture into politics and reinstated intentional coherence to Mao’s purpose.

This article argues that both mainstream narratives, exemplified by MacFarquhar, and alternative interpretations, including those of Russo and Andreas, are shaped by a shared methodological limitation: they interpret the early Cultural Revolution retrospectively, reading its formative moments through the lens of later outcomes—most notably the mass upheaval of late summer 1966. In doing so, they impose a teleological structure that flattens the discontinuities and uncertainties. Much of the scholarship on the Cultural Revolution’s origins and early trajectory has relied on retroactively assembling a linear sequence that appears to culminate inevitably in mass mobilization. By centering on Mao’s presumed fixed intentions, these accounts impose causal coherence, projecting an illusion of inevitability onto a complex, contingent historical process. A more historically grounded understanding requires unsettling these retrospective causal chains and reconstructing the context-specific dynamics through which events actually unfolded.

The remainder of the article reexamines the ideological and cultural struggles that preceded and shaped the early Cultural Revolution, offering a reinterpretation of the movement’s origins and early formation. Contrary to prevailing accounts, these struggles were not a calculated prelude to a broader political assault on the party-state. The movement instead began as a militant cultural upheaval that must be understood in its original historical context and on its own discursive terms, as integral to Mao’s revolutionary project rather than a calculated instrument in the service of preconceived political objectives. This article, therefore, calls for renewed attention to the cultural and ideological stakes of the early Cultural Revolution, before they were drawn into—and eventually overtaken by—the mass insurgency that retrospectively came to define the Cultural Revolution.

To distinguish the early “cultural revolution” from the later mass-revolutionary upheaval is not to posit an ontological separation between culture and politics. Cultural struggle in Mao’s China was inseparable from questions of authority and power. The distinction drawn here operates at the level of historical form: It concerns how political contestation was structured and authorized at different moments in 1966. In late spring and early summer of that year, the campaign, despite its radical rhetoric, functioned as a militant cultural rectification. It was directed primarily at the cultural and educational establishment and remained largely confined to intra-party channels, operating through established mechanisms of party oversight and organizational discipline. Only later did the movement assume the form of decentralized mass insurgency that openly challenged party hierarchy and authority. The issue here, therefore, is not whether culture was political but how a bounded campaign of cultural rectification was transformed into a revolutionary upheaval that exceeded its original institutional and organizational limits.

## Mao’s Cultural War: The Cultural Origins of the Cultural Revolution

In November 1965, Yao Wenyuan published a polemical article accusing Wu Han—author of *Hai Rui Dismissed from Office*, historian, and vice mayor of Beijing—of “anti-party and anti-socialist” transgressions. Often read as an early salvo in Mao’s political maneuvering against the Beijing municipal leadership headed by Peng Zhen—and, by extension, against senior party leaders such as Liu Shaoqi—this episode has commonly been folded into a narrative of strategic escalation. Mao, in the words of MacFarquhar and Schoenhals, “had bigger fish to fry than Wu Han” (MacFarquhar and Schoenhals, 2006: 17). While a full reassessment of the episode lies beyond the scope of this article, it is nevertheless crucial to reposition Yao’s intervention within the radicalizing ideological and cultural milieu of the mid-1960s—an environment that shaped not only the political resonance of the *Hai Rui* affair but also the directions in which it was subsequently pushed.

A junior propaganda official and radical intellectual based in Shanghai, Yao gained notoriety as the “bludgeon” 棍子 in China’s academic and literary world. Since the late 1950s, he had built his reputation through a series of sharply polemical articles targeting prominent cultural figures and their works, positioning himself as a key enforcer of ideological orthodoxy (Shi, 2012: 165–276). His attack on *Hai Rui* followed this established pattern of ideologically driven scrutiny. Yet, when viewed within the broader context of ideological and cultural radicalization of the mid-1960s, Yao’s article—aside from the political upheaval it helped unleash—was not, in itself, exceptional.

This radicalization had deep roots. In the early 1960s, as the CCP retreated from radical economic policies, it briefly adopted a more conciliatory cultural stance (Goldman, 1969; Luo, 2008: 185–262). However, in late 1962, Mao launched a counteroffensive against perceived ideological laxity, warning that class enemies were exploiting economic setbacks to undermine socialism, reaffirming the primacy of ideological struggle (Documentation Research Office, 2003: 2.1254–55). From that point onward, the party’s cultural and ideological policies became increasingly militant. Criticism intensified across literature, film, theater, philosophy, historiography, and education, with class struggle becoming the dominant lens through which intellectuals and their works were scrutinized. By the mid-1960s, this drive had reached its most punitive phase, dealing particularly severe blows to literary works and historical scholarship (Dai, 1994; Goldman, 1973). *Hai Rui* became trapped in this broader wave of militant cultural criticism. In this context, Yao Wenyuan’s 1965 attack on Wu Han did not fundamentally differ in style or substance from contemporary ideological campaigns; what set it apart was the far-reaching political consequences it unleashed.

### “The East Wind Must Overwhelm the West Wind”: Transforming the Cultural Realm

The cultural, literary, and intellectual spheres were among the most dynamic and fiercely contested arenas of China’s tumultuous twentieth century. Mao’s enduring commitment to radical cultural change can be traced to his formative years in the May Fourth era, a moment of intellectual ferment that left an indelible mark on his outlook. As Meisner observed, the idea of cultural revolution was not Mao’s invention but emerged from broader intellectual currents in early twentieth-century China. Well before the Cultural Revolution of 1966, which Meisner aptly termed “China’s second cultural revolution” (Meisner, 1985: 286), the New Culture and May Fourth movements had already launched uncompromising attacks on China’s entrenched traditions, casting the demolition of Confucian orthodoxy as the precondition for national regeneration. For these early radicals, as for Mao later, China’s predicament was fundamentally cultural: An intellectual class bound by stale convention and estranged from the people appeared to be both symptom and cause of stagnation (Chow, 1960; Lin, 1978). This formative logic profoundly shaped Mao’s lifelong preoccupation with radical cultural change and with the fraught relationship between intellectuals and the masses. Mao’s subsequent political experience—forged in war and revolution—only deepened his conviction that genuine change required not merely the seizure of state power but an ongoing struggle to remake culture and consciousness.

During wartime, the CCP’s vision of radical cultural transformation struggled to take hold. The Sino-Japanese War of the late 1930s, however, brought an influx of educated urban youth—many from bourgeois backgrounds—to Yan’an, the CCP’s remote headquarters. Viewing these intellectual newcomers as ideologically suspect, party leaders sought to re-educate them through revolutionary struggle, reinforcing the principle that culture and art must serve as tools of mass political mobilization. Mao’s 1942 “Talks at the Yan’an Forum on Literature and Art” articulated this vision. Declaring that “literature and art should serve the workers, peasants, and soldiers,” Mao called on intellectuals to undergo self-reform, to integrate with the masses, and to produce works reflecting a revolutionary perspective, insisting that their subjective consciousness be transformed through physical labor and political engagement (Judd, 1985: 377–408). This ideological framework informed CCP cultural policies both during the war and the early PRC years.

After the CCP took national power in 1949, it continued to draw upon the principles forged during the Yan’an years. The transition to socialism, however, posed urgent and unresolved questions: What ideological and cultural transformations should accompany far-reaching economic change? How was socialist culture to be defined, and how far could revolutionary values shape its development? What role were intellectuals to play in a society that claimed to be led by the working masses? And how were the tensions between cultural inheritance, destruction, and creation to be negotiated?

The promotion of a new, distinctly revolutionary culture remained a key priority of the CCP throughout the Mao era. By the 1950s, the term “cultural revolution” gained increasing prominence, initially denoting efforts to popularize culture among working people, expand literacy, and advance scientific knowledge. At a 1956 conference, Mao articulated this vision: “We need to develop science, to eliminate ignorance and stupidity, and to revolutionize technology—we call this cultural revolution” (Documentation Research Office, 2013: 2.515). But by the late 1950s, the concept and language of cultural revolution had grown increasingly militant. Though the Great Leap Forward is primarily remembered for its reckless economic policies, it also encompassed ambitious cultural initiatives. Officially inaugurated in 1958, what was explicitly termed a “cultural revolution” 文化革命 pursued several interrelated objectives: integrating intellectuals with the masses to ensure cultural production would be both accessible and socially relevant; democratizing cultural creation by encouraging workers and peasants to engage in artistic and intellectual activities; and aligning cultural work with political struggle, rendering it an instrument of party policy. A *People’s Daily* editorial on June 9, 1958, evocatively titled “Cultural Revolution Has Begun” 文化革命开始了, described the campaign as a “movement for the cultural emancipation of all working people” (Renmin ribao, June 9, 1958). On September 23, 1958, Minister of Propaganda Lu Dingyi identified its twin objectives: eradicating cultural deprivation among workers and peasants, and purging bourgeois ideas among intellectuals (Renmin ribao, September 23, 1958). Framed in these terms, the cultural revolution of the Great Leap Forward sought simultaneously to broaden mass participation and to reconfigure the cultural and ideological landscape in service of the socialist state.

Sheila Fitzpatrick’s seminal work on Soviet history provides a productive comparative lens for understanding the shifting cultural dynamics of Mao’s China. Fitzpatrick differentiates between Lenin’s Cultural Revolution, oriented toward expanding popular literacy and integrating technical expertise, and the militant Cultural Revolution of the early Stalin era, which sought to aggressively proletarianize intellectual life, purge bourgeois influences, and align cultural practices with party doctrines (Fitzpatrick, 1978). A broadly comparable trajectory can be discerned in China during the late 1950s and early 1960s. While both Lenin and Mao viewed cultural transformation as essential to socialist development, Mao’s iconoclastic vision moved in a more radical direction, casting the eradication of non-socialist ideas and values as a prerequisite for revolutionary change (Meisner, 1999: 296–98). Initially, the CCP pursued a relatively moderate strategy, emphasizing education, ideological cultivation, and mass participation. Yet this civilizing project—articulated in the language of communist modernity—soon gave way to an uncompromising cultural class war, a reorientation that closely parallels the Soviet turn of the late 1920s.

This ideological hardening accelerated after 1962, as the CCP reoriented its priorities toward class struggle on two parallel fronts: the Four Cleanups—later renamed the Socialist Education Movement—which targeted grassroots cadres, and a renewed ideological offensive in the cultural sphere. During this period, Mao grew increasingly alarmed by what he perceived as pernicious trends in literature, academia, and education, repeatedly warning that these sectors had become arenas of ideological deviation and “revisionism.” His anxiety—shaped by his reading of Khrushchev’s “restoration” in the Soviet Union and reinforced at home through the Socialist Education Movement’s broad program of cadre rectification and political re-education—deepened his sense that the party was confronting a growing crisis of ideological direction.^3^

Against the background of this intensifying ideological climate of class struggle and revisionist anxiety, Mao—during a tour of eastern China in December 1962—met with provincial leaders and linked the problem of traditional theater to the broader ideological struggle. He observed, “The theatrical stage is filled with stories about emperors and generals, talented scholars and beautiful ladies 帝王将相, 才子佳人—this has led to a situation where the ‘Western wind’ is overpowering the ‘Eastern wind.’ But the Eastern wind must prevail” (Documentation Research Office, 2013: 4.177). By invoking the metaphor of contending winds, Mao cast the predominance of feudal and bourgeois themes in theater as a symptom of ideological backsliding. He reiterated these concerns at a central conference on September 27, 1963, invoking the slogan “discard the old to make way for the new” 推陈出新 and emphasizing the imperative to eradicate feudal and capitalist influences in favor of socialist content. Cultural transformation, Mao argued, was essential to ensuring that the superstructure kept pace with the socialist economic base (Documentation Research Office, 2013: 5.263–64). These criticisms sharpened further in November 1963, when Mao singled out the Ministry of Culture for ideological negligence, suggesting that, if it failed to reform, it might better be renamed the “Ministry of Emperors and Generals,” the “Ministry of Scholars and Beauties,” or even the “Ministry of Dead Foreigners” (Documentation Research Office, 2013: 5.285).

In the early and mid-1960s, internal reports documented a wide range of politically undesirable social customs and folk beliefs. A report from rural Guangdong, for example, recorded the construction of temples, altars, and ancestral halls, and observed that activities such as worshiping deities and ancestors, festival performances, and communal feasting seriously disrupted collective production. Taoist priests, shamans, and geomancers offered services ranging from fortune-telling and spirit-summoning 请仙 to geomancy consultations and the exorcism of ghosts and demons.^4^ Such practices were not confined to the countryside. Reports indicated that so-called “feudal superstitions” had also spread into industrial enterprises and urban settings. In Hebei province, it was reported that many employees in state-owned enterprises were engaging in physiognomy 相面 and fortune-telling. At one enterprise, more than 70 percent of the workforce had consulted divination, including nearly half of the CCP and Communist Youth League members. In some coal mines, party cadres led workers in constructing shrines for the “mine god” 窑神爷, organizing lavish ritual feasts, and performing ceremonies for divine protection.^5^ Urban youth were likewise depicted as deeply entangled in religious practice. Reports noted participation in worship of deities, church attendance, baptism, and even aspirations toward ordination as Taoist priests. Fortune-telling proved especially popular, as young workers and students sought guidance on educational prospects, career opportunities, and romantic relationships.^6^

In addition to engaging in “superstitious” practices, young people were said to be enamored with “vulgar music” 黄色音乐, romantic love songs, social dancing, and other forms of “lowbrow entertainment” 低级趣味. One report noted that the *Selected Collection of Foreign Songs*, published by the Music Publishing House, had sold out rapidly, being especially popular among young students and workers. The collection included songs from the Soviet Union, Eastern Europe, the United States, Britain, France, and Japan, many of which were allegedly reflective of “bourgeois tastes and lifestyles.”^7^ Another report described the popularity of social dancing in factories, schools, and government offices, where events were said to feature “vulgar music,” dim lighting, and “suggestive or obscene movements.” Brawls reportedly broke out as young men fought over female partners, and many youths were said to spend most of their leisure time dancing, leading to failure to meet production targets and declining participation in ideological study.^8^ According to yet another report, “obscene, absurd, and reactionary literature” circulated widely among urban youth through private lending, hand copying, and renting.^9^ Pamphlets depicting sexual behavior and “decadent lifestyles” were said to be especially common; among those reading such materials, 10 percent were young workers, 15 percent middle school students, and 20 percent unemployed youth.^10^

To Mao, such reports appeared to confirm that ideological disorder had seeped into everyday life, deepening his fear that the cultural sphere was becoming a primary locus of “revisionist” danger. This perceived disorder was increasingly blamed for the spiritual emptiness and moral decay among China’s young generation, prompting CCP leaders—above all Mao—to elevate the role of literature and art in constructing and disseminating revolutionary culture. On December 12, 1963, after reviewing a report on Ke Qingshi, party chief of Shanghai, who was personally overseeing socialist literature and art to reclaim everyday cultural life, Mao issued a pointed critique. Despite the transformation of the socioeconomic base, he argued, the ideological superstructure, particularly literature and the arts, remained largely unmolded and deeply problematic (Documentation Research Office, 1996a: 437). Mao forwarded the report, along with his critical remarks, to Peng Zhen, executive secretary of the CCP’s Central Secretariat and party chief of Beijing, underscoring the urgency of promoting revolutionary culture as a political task.

In a speech delivered on February 13, 1964, Mao sharply criticized intellectuals and cultural workers for their detachment from the realities of the new socialist era. He declared, “We need to drive out the opera performers, poets, writers, and playwrights from the cities and send them all down. They should all be sent to the countryside and factories.”^11^ Reiterating these concerns on May 13, Mao condemned the persistent elitist orientation of cultural production. “Hasn’t it been advocated for many years that literature and art should serve the workers, peasants, and soldiers? Everyone talks about it, but when it comes to staging operas, it’s still about emperors and generals. Those people still don’t go down to the countryside or factories” (Documentation Research Office, 2013: 5.350). Two days later, Mao again targeted the literary and artistic community, criticizing its fixation on “the dead people—if not the dead people, then the foreigners, and the foreigners are also dead.” He lamented the persistence of “old culture,” insisting that it be replaced with revolutionary alternatives. “Many aspects of our society’s ideology remain unreformed. Old ideologies are incredibly resistant to change. . . . So, we must drive them out.”^12^

On June 27, 1964, Mao delivered his harshest criticism to date of culture workers, responding to a report from the Central Propaganda Department on “class struggle” in cultural circles.^13^ He condemned cultural workers for failing to implement party policy, accusing them of elitism and of estrangement from the working people. Their growing ideological laxity, he warned, had pushed them toward revisionism and, if left unchecked, could transform them into a counterrevolutionary force (Documentation Research Office, 2013: 5.367–68). This denunciation was subsequently formalized in a Party Center document and circulated throughout the CCP bureaucracy, triggering a sweeping ideological rectification campaign within the Ministry of Culture. The campaign led to extensive personnel purges, making the ministry the first major institutional casualty of the radical cultural movement (Zhao, 2005: 26–27). At this moment, Yao Wenyuan’s criticism of Wu Han’s *Hai Rui* play—later widely regarded as the Cultural Revolution’s opening strike—was still in preparation.

In response to Mao’s repeated criticisms, other top leaders swiftly echoed his concerns. On January 3, 1964, Liu Shaoqi convened a central conference on cultural and literary policy, attended by more than forty senior officials, including Deng Xiaoping, Peng Zhen, Zhou Yang, Chen Boda, and Kang Sheng. At the meeting, Peng Zhen acknowledged that revolutionary efforts in Beijing’s cultural spheres had been inadequate. Liu Shaoqi lamented that, after fourteen years of socialist revolution, the proletariat had secured only limited ground in the cultural arena, which he argued remained dominated by feudal and capitalist influences. Deng Xiaoping characterized literature and art as key battlegrounds in the superstructure, warning that “counterrevolutionary restoration may start here.” Deng outlined three measures to address the crisis: (1) unifying ideological understanding, (2) formulating a strategic plan, and (3) assembling capable teams.^14^

Liu Shaoqi’s language sharpened further at a July 1964 meeting, where he accused cultural workers of disseminating ideas that undermined the socialist state, likening such practices to facilitating capitalist restoration. He warned that party authority remained fragile across cultural institutions and schools, estimating that more than one-third operated beyond the party’s effective control. Rectifying this condition, Liu insisted, would require not a single, decisive campaign but a sustained, recurring revolutionary process, involving successive rounds of intervention in the years ahead.^15^

By the mid-1960s, Mao had come to regard the party’s ideological and cultural work as increasingly ineffectual, a view that helped propel events in a more radical direction. Despite the party’s extensive control over mass media, education, and cultural production, skepticism and disillusionment remained widespread in the aftermath of the Great Leap Forward’s catastrophic failure. The party-state’s ideological apparatus struggled to sustain revolutionary enthusiasm, and Mao grew increasingly frustrated. This diagnosis mirrors what Peter Kenez and David Brandenberger identify in Soviet Russia: the moment when a ruling party, confronted with a “propaganda state in crisis,” interprets ideological weakness not as a problem of communication but as a political emergency demanding systematic corrective measures (Brandenberger, 2012; Kenez, 1985). In China, Mao’s growing dissatisfaction similarly encouraged a shift toward a more militant cultural strategy. While the CCP leaders might have diverged in rhetoric, strategy, and emphasis, they largely converged on the view that forceful interventions were necessary to reassert ideological authority.

These initiatives were already underway by July 1964, when Peng Zhen presided over a Central Secretariat meeting to convey Mao’s concerns. Citing Mao, Peng warned that if left unchecked, this could lead to a “Petőfi Club” scenario—referring to the Hungarian intellectual forum linked to the 1956 uprising—and raise the specter of “counterrevolutionary restoration.” In response to Mao’s directive, the meeting established the Cultural Revolution Five-Person Group, comprising Peng Zhen, Lu Dingyi, Kang Sheng, Zhou Yang, and Wu Lengxi. As a precursor to the Central Cultural Revolution Group (hereafter CCRG) during the Cultural Revolution, this body was tasked with implementing Mao’s vision for literature, the arts, and academic and cultural work (Editorial Group of Biography of Peng Zhen, 2012: 342–43). At this stage, the Five-Person Group represented Mao’s attempt to strengthen—not yet circumvent—the party’s existing bureaucratic functions by creating an intra-bureaucratic mechanism for exerting direct influence over ideological and cultural affairs. Yet Mao again grew dissatisfied with its limited effectiveness. By mid-1966, his frustration culminated in a profound rupture with the party apparatus, marking his turn toward a far more radical, activist approach to cultural and ideological matters.

### “We Don’t Have Our Intellectuals”

Mao’s intensifying emphasis on cultural class struggle rested on two interrelated assessments: his reading of developments within the cultural sphere and his deepening distrust of intellectuals’ political reliability after the late 1950s. Such skepticism, however, was hardly unique to Mao. Anti-intellectualism had been a persistent undercurrent within the Chinese communist movement since its inception, even as many of its leaders themselves emerged from elite educational backgrounds. As Eddy U observes, the relationship between Chinese communism and intellectuals was both mutually constitutive and deeply fraught, marked by a persistent ambivalence between reliance on intellectual expertise and suspicion of bourgeois predispositions (U, 2019). After 1949, the CCP sought to incorporate the educated elite into the socialist order through a dual strategy of inclusion and ideological remolding. This pragmatic accommodation initially acknowledged intellectuals’ indispensability to socialist modernization, but over time, distrust and political discipline increasingly displaced accommodation, hardening the ideological stakes of cultural work (Cheek, 2016: 70–216).

This underlying ambivalence acquired new urgency in the early 1960s, as the party moved to restore institutional order after the disruptions of Great Leap Forward populism—a shift that unfolded alongside what Meisner termed a “bureaucratic restoration” (Meisner, 1999: 245). This turn placed renewed stress on administrative regularization and professional standards across higher education, scientific research, and industry, strengthening the authority of specialists and restoring credentialed expertise to the center of institutional life (MacFarquhar, 1997: 72–120). For Mao, this technocratic consolidation revived the epistemic and social hierarchies he regarded as politically suspect, reinforcing his belief that the cultural and educational spheres required more radical ideological remaking.

Despite his own considerable erudition, Mao consistently harbored a deep mistrust of China’s educated elite. In the 1950s, his critiques of intellectuals remained primarily ideological, censuring them for their bourgeois tendencies and social estrangement from the masses. By the mid-1960s, however, this suspicion had sharpened: he increasingly viewed the pre-revolutionary backgrounds of many intellectuals not simply as vestiges of the *ancien régime* but as active political liabilities, capable of undermining the socialist present.

On November 26, 1963, in a meeting with Cuban cultural officials, Mao asserted that China’s schoolteachers and university professors remained essentially products of the old society, emphasizing that their ideological transformation would be a protracted process—if it was achievable at all.^16^ Mao reiterated these concerns on August 25, 1964, in a discussion with a delegation of African and Latin American students. He argued that intellectuals would be of little value unless they integrated with the working masses and singled out Peking University as emblematic of entrenched elitism and ideological impurity.^17^ Days later, at a meeting with a Laotian cultural delegation, he noted that the cultural sector had inherited hundreds of thousands of bourgeois intellectuals, including professors, teachers, artists, singers, painters, and filmmakers, warning that the question of whether the proletariat would triumph over the bourgeoisie remained unresolved. Admitting the party’s past negligence in this area, he called for a rectification campaign to reform these intellectuals, adding bluntly: “If that makes those people sleepless for a year or two, that will make me incredibly happy.”^18^

Mao’s anxieties about the cultural sectors, particularly the intellectuals, were not mere paranoia. In the mid-1960s, the party leadership increasingly emphasized class struggle, a message that intensified as it filtered down the party hierarchy. Eager to align with this political line, local officials inundated the central leadership with reports portraying class struggle as “exceptionally acute” in their regions. Against the backdrop of the Great Leap Forward’s catastrophic failures, internal reports documented pervasive disorder across cultural, educational, and social institutions. This steady flow of dire assessments reinforced Mao’s belief that ideological threats were mounting, deepening his sense of crisis. For example, reports from Mao’s home province of Hunan depicted the cultural and educational sectors as key battlegrounds in the struggle against ideological subversion. Schools were rife with counterrevolutionaries, rightists, and bourgeois intellectuals who sought to subvert socialism (Dai, 2005: 33, 47–49). One investigation claimed that half of Hunan’s 140,000 primary and secondary school teachers came from exploiting-class backgrounds or bore significant political stains. Many were accused of harboring “a hatred that cuts to the bone” 刻骨仇恨 for the party, opposing the socialist system, and perpetuating reactionary attitudes.^19^ In April 1964, Hunan’s provincial leadership made purging the teaching staff a priority of the Socialist Education Movement, issuing a directive outlining thirty categories of politically undesirable individuals, including former landlords, rich peasants, capitalists, counterrevolutionaries, secret-society members, monks, nuns, and others deemed threats to the socialist order (Party History Research Office of the Hunan Provincial Committee of the Chinese Communist Party, 2009: 397–401). Comparable efforts to purge cultural and educational institutions were initiated nationwide, reflecting broader anxieties about political and ideological reliability.^20^

Mao’s dissatisfaction with the cultural and intellectual spheres had become increasingly pronounced by late 1964. In November, he openly questioned the party’s grip on the cultural field, asking, “How much of the cultural sector is in our hands? Twenty percent? Thirty percent? Or half? Or is most of it not under our control? I believe at least half is beyond our control” (Bo, 1993: 1227). The following month, at a meeting with visiting Asian and African writers, he reiterated his frustrations. While acknowledging the necessity of retaining intellectuals from the pre-revolutionary era—“otherwise, we would have no intellectuals, no professors, no schoolteachers, no journalists, and no artists”—he nonetheless dismissed them as ideological adversaries: “Those people cling to their own old ideas and reject ours. I call them the bad people.”^21^ In August 1965, during a conversation with French Minister of Cultural Affairs André Malraux, Mao once again underscored his anxieties about the residual influence of China’s intellectual class. Reflecting on the composition of “revisionist” elements within Chinese society, Mao observed, “This group includes former landlords and wealthy peasants, former capitalists, intellectuals, journalists, writers, artists, and some of their descendants.” Pressed on why writers and artists were included among these suspect groups, he was unequivocal: “We inherited them from the old society. We had no artists, journalists, writers, professors, or teachers of our own; they were all remnants of the Kuomintang.”^22^

On the eve of the Cultural Revolution, Mao intensified his attacks on intellectuals and the educational and cultural spheres. On April 14, while commenting on a report about universities experimenting with a “half-work, half-study” system, he sharply rebuked professors and students for their preoccupation with book learning and ignorance of practical concerns such as warfare, revolution, industry, and agriculture. “Many of them have only one skill—opposing the Communist Party, the people, and the revolution,” he declared, and added, “That’s why I often say that intellectuals, compared to workers and peasants, are the most ignorant people.” To remedy this, he urged relocating schools to factories and rural areas, enforcing the half-work, half-study model, and shutting down any that refused to comply (Documentation Research Office, 1998a: 34–35). His concerns resurfaced on May 5 during a banquet for Albanian visitors. There, he warned that the CCP had absorbed numerous bourgeois intellectuals, while university students remained largely from bourgeois or landlord families. Some of these “old people” 旧人, he alleged, had even infiltrated the party, lying in wait to strike.^23^ Two days later, in what became famously known as the May 7 Directive, Mao outlined his vision for a revolutionary society in a letter to Lin Biao. Rejecting elite-oriented education, he called for a radical overhaul: formal schooling must be shortened, and education must integrate academic study with ideological work, manual labor, mass mobilization, and military training. “The domination of our schools by bourgeois intellectuals must end,” he declared (Documentation Research Office, 2013: 5.585). For Mao, such “bourgeois” intellectuals represented the remnants of the old society and the persistent source of cultural dysfunction in socialist China, a threat that, in his view, demanded a thoroughgoing assault on the country’s educational and cultural institutions.

## Competing Cultural Revolutions

Mao’s views on China’s literary and cultural spheres were closely intertwined with those of his wife, Jiang Qing. Her uncompromising identification with Mao’s cultural vision positioned her as both a trusted confidante and an increasingly consequential figure within the radical cultural milieu of the mid-1960s. In a 1967 speech, Jiang Qing presented herself as Mao’s “roving sentinel” in culture, literature, and art. More significantly, she offered a sweeping accusation of China’s cultural and educational institutions, denouncing their entrenched elitism and ideological deformation: “In literature and art, there is an overwhelming emphasis on foreign and antiquarian themes. The representation of workers, peasants, and soldiers is often distorted. As for schools, they are almost entirely dominated by bourgeois intellectuals and revisionist ideas” (Jiang, 1967: 228, 233–34).

Jiang Qing’s transition from operating behind the scenes to assuming a prominent political role began within the cultural sphere. On June 23, 1964, she emerged as a leading force in theatrical and artistic reform by delivering a speech at a symposium on modern-style Peking Opera convened by Zhou Enlai. This marked her first public address since her arrival in Yan’an in 1937. In her remarks, she denounced the prevailing theatrical and artistic landscape: “The theater, a place to educate the people, is now dominated by emperors, generals, gifted scholars, and beautiful ladies—this is all feudalistic and bourgeois stuff. On the stage of our socialist homeland, led by the Communist Party, the principal characters are not workers, peasants, and soldiers, the creators of history and masters of this country. This is inconceivable” (Jiang, 1967: 10). Mao endorsed Jiang Qing’s speech, remarking that it was “well spoken” 讲得好 (Documentation Research Office, 1996b: 89).

With Mao’s backing, Jiang Qing rose to prominence as a key advocate of revolutionary art, leading efforts to reclaim cultural spaces denounced as overgrown with “poisonous weeds.” Beginning in 1963, she played a pivotal role in reforming traditional Peking Opera, laying the groundwork for the “model operas” that would later define Cultural Revolution–era aesthetics. Scholars of Cultural Revolution cultural production—most notably Barbara Mittler and Paul Clark—have challenged the prevailing view that this period marked an abrupt, wholly negative rupture, arguing that Cultural Revolution culture formed an integral part of a long continuum of artistic experimentation and cultural modernization in twentieth-century China (Clark, 2008; Mittler, 2013). Positioned at the forefront of this militant cultural initiative, Jiang Qing’s campaign to transform Peking Opera and the broader cultural landscape was hailed by official media in 1967 as the “great beginning of the Great Proletarian Cultural Revolution” (Hongqi, May 6, 1967).

It was in this context of militant cultural remaking that the *Hai Rui* incident took on its explosive significance. Orchestrated by Jiang Qing with Mao’s backing, the late-1965 *Hai Rui* incident is widely regarded as the “opening shot” of the Cultural Revolution. Yet the critical question remains: what kind of “cultural revolution” did it catalyze? Addressing this question requires close examination of two key texts produced in February 1966: the “Report Outline to the Center by the Group of Five” (known as the February Outline) and the “Summary of the PLA Forum on Literature and Art Work” (hereafter the Summary). Both were programmatic interventions intended to guide ideological and cultural work, yet they advanced sharply divergent approaches, revealing the emergence of competing cultural revolutions within the party leadership.

The February Outline emerged amid the escalating controversy surrounding the *Hai Rui* affair. After Yao Wenyuan’s criticism of the play, top CCP leaders were initially slow to recognize its broader political implications. Most major newspapers in Beijing and across the country declined to reprint Yao’s article, provoking Mao’s displeasure. In response, from December 1965 to January 1966, a wave of sharply worded essays targeting Wu Han and other prominent intellectuals signaled an intensifying ideological assault. In early February, Peng Zhen convened the Cultural Revolution Five-Person Group to codify the party’s approach to academic criticism, resulting in the drafting of the February Outline. Approved at a Politburo meeting presided over by Liu Shaoqi, the document was positioned as the CCP’s authoritative stance on ideological struggle. Peng briefed Mao on February 8, and Mao raised no conspicuous objections. By February 12, the February Outline had been disseminated nationwide, consolidating its status as the guiding framework for ideological criticism in academic and cultural spheres.^24^

The February Outline endorsed, in principle, the wave of ideological criticism that was spearheaded by the *Hai Rui* affair. At the same time, it urged restraint and procedural discipline. Emphasizing party leadership, organizational discipline, and prudence, it called for a policy of “letting go” 放—a hallmark of the Hundred Flowers Movement in 1957—to encourage diverse opinions. It stressed reliance on factual evidence, scholarly argumentation, and principles such as “seeking truth from facts” and “equality for all in the pursuit of truth.” The document also warned against authoritarian tendencies, particularly the dominance of “academic despots” 学阀, and urged moderation in public criticism. Notably, even self-identified “leftist” critics such as Yao Wenyuan were expected to engage in self-criticism to guard against dogmatism.^25^ By elevating scholarly rigor and organizational supervision over ideological militancy, the February Outline aimed to confine radical cultural attacks within clearly defined bureaucratic limits.

The Summary, drafted under Jiang Qing’s supervision around the same time, articulated a starkly different approach. In February 1966, with Mao and Lin Biao’s backing, Jiang convened a meeting on the military’s role in literature and art. According to General Liu Zhijian, deputy director of the PLA’s Political Department, the meeting featured film and theatrical screenings, as well as over a dozen discussions in which Jiang elaborated on her cultural views and launched scathing attacks on existing works. Declaring that “our literary and artistic circles are in disarray,” she decried the dominance of “emperors, generals, literati scholars, beauties, foreigners, and the dead” on the stage. The cultural sphere, she warned, had been seized by a “black anti-party and anti-socialist line.” Jiang detailed her efforts to root out ideological deviations in Beijing opera, lamenting that Mao’s instructions on cultural matters had been ignored. “The current debates are merely preliminary skirmishes,” she claimed, “the decisive battle has yet to come” (Liu, 1996: 209–10). Drafted from meeting minutes, the Summary underwent multiple revisions by Chen Boda and Zhang Chunqiao, with Mao himself amending it on three occasions.^26^

The Summary repeatedly invoked terms such as “cultural revolution” and “socialist cultural revolution,” citing them fourteen times alongside variants such as “socialist revolution on the cultural front.” Across these formulations, the Summary framed cultural work as a site of acute ideological confrontation, emphasizing the “struggle between two classes” and the “struggle between two lines.” Although phrased differently, these expressions collectively portrayed the cultural realm as a battleground for class war, in sharp contrast to the February Outline’s more restrained approach.

As a programmatic manifesto for a radical cultural revolution, the Summary repudiated post-1949 literary and artistic developments, asserting that the cultural sphere had fallen under an “anti-party, anti-socialist black line” dominated by bourgeois and revisionist ideas. This ideological threat, it insisted, demanded a “resolute socialist revolution on the cultural front” to eradicate bourgeois influence. Celebrating Jiang Qing’s leadership, the document presented recent developments in literature and art—above all, the rise of revolutionary Peking Opera—as concrete proof that an uncompromising “socialist cultural revolution” was already underway. This revolution, aimed at remaking China’s cultural order, was to be “carried through to completion.” The claim that such a cultural revolution had begun to show results only over the previous three years was, in fact, a tribute to Jiang Qing, who was portrayed as having succeeded where the existing leadership had failed. As MacFarquhar observes, the Summary thus did more than articulate a new cultural line: it effectively constituted a rival cultural headquarters, positioned against Peng Zhen and his institutional authority (MacFarquhar, 1997: 452). On April 10, 1966, the Party Center circulated the Summary nationwide. Together with the subsequent May 16 Notice (discussed below), the Summary cleared the ground for a far more radical intervention in the cultural sphere. Though “culture” remained a fluid and multifaceted concept, the notion of “cultural revolution” now acquired renewed political salience, activated through a coordinated set of initiatives aimed at eradicating allegedly feudal and bourgeois influences while constructing a socialist, revolutionary culture.

Shortly after the release of the Summary, the *PLA Daily* published a pivotal editorial on April 18 titled “Raise High the Great Red Flag of Mao Zedong Thought and Actively Participate in the Socialist Cultural Revolution.” Reprinted in *People’s Daily*, this editorial served as an unequivocal call to arms for the impending Cultural Revolution, drawing directly on the themes and formulations of Jiang Qing’s Summary. It condemned the so-called “black line”—a metaphor for bourgeois ideology opposed to Mao Zedong Thought, the party, and socialism—and demanded a “great cultural revolution” 文化大革命. In the weeks that followed, the *PLA Daily*—along with other national and regional papers—intensified the campaign, publishing a torrent of articles targeting prominent intellectuals both within and outside the party. These pieces accused them of attempting to “restore capitalism” and exhorting the masses to “open fire on the anti-party, anti-socialist black line” in the cultural sphere. At the same time, they called for the systematic promotion of proletarian values across academia, education, literature, and the arts. Framing the Cultural Revolution as a protracted struggle lasting decades or even centuries, these publications—through editorials, public forums, and purported letters “from the masses”—amplified attacks on ideological adversaries and helped prepare the terrain for the militant upheaval soon to engulf China’s cultural and intellectual landscape.

## Counteroffensive: “Our Cultural Revolution Is About to Begin”

In the spring of 1966, Mao increasingly invoked the language and concept of “cultural revolution.” In March, he revised the Summary submitted by Jiang Qing, appending a paragraph in which he declared the need for a “great socialist revolution on the cultural front” to eradicate the bourgeois “black line.” He warned that even after its eradication, new manifestations of bourgeois ideology would inevitably reappear, necessitating continuous vigilance and struggle (Documentation Research Office, 2003: 2.1403).

From March 17 to 20, 1966, Mao convened a Politburo meeting in Hangzhou to assess the “current situation of cultural revolution” (Wu, 1999: 937). There, he adopted a markedly more militant tone, signaling a clear departure from the ambiguity he had shown toward the *Hai Rui* affair only weeks earlier. Declaring that “a great cultural revolution must be launched” 要搞文化大革命, he called for a comprehensive assault on China’s intellectual and cultural establishment. He contended that while the post-1949 state had initially adopted a relatively inclusive posture toward intellectuals, the academic and educational sectors had since fallen under bourgeois domination. “The deeper the socialist revolution goes, the more resistance they offer,” he warned, sharply rebuking party leaders for their complacency. The limited penetration of Marxist discourse in fields such as literature, history, philosophy, law, and economics, he lamented, was symptomatic of a broader ideological stagnation. Mao pressed party leaders at all levels to scrutinize who exercised effective control over schools, newspapers, magazines, and publishing houses, and stressed the urgent need to criticize bourgeois academic authorities and foster a new generation of politically reliable Marxist scholars.^27^

On March 18, Mao addressed the absence of effective political leadership in literature, arts, and education, underscoring the imperative to dismantle “bourgeois authorities” (Documentation Research Office, 2013: 5.567). Two days later, he extended this critique, indicting the party’s longstanding failure to recognize bourgeois dominance in the cultural sphere and asserting that primary, secondary, and higher educational institutions remained controlled mainly by individuals from former exploiting classes. Rectifying this condition, he insisted, would require five to ten years of sustained struggle, not a short-term campaign (Documentation Research Office, 2013: 5.568–69).

To this end, Mao outlined two key tasks: first, delegitimizing bourgeois intellectual authority through sustained, relentless criticism; and second, promoting physical labor and mixed work-study programs as key instruments of ideological remolding (Documentation Research Office, 2003: 2.1405). He further stressed the critical role of the younger generation in advancing ideological criticism and propelling a militant cultural revolution. Encouraging students to challenge authority, he declared, “Do not suppress young people; just let them emerge.” Mao called for empowering students, teaching assistants, and junior faculty while isolating senior professors resistant to ideological transformation. He went so far as to argue that younger and less credentialed individuals should be permitted to overturn their senior and more established counterparts, a reversal he viewed as essential to breaking the grip of entrenched intellectual elites. Citing figures such as Yao Wenyuan and Qi Benyu as exemplars, Mao projected the emergence of a new revolutionary cohort—young, politically hardened, and ideologically resolute. He insisted that “propaganda ministers must not suppress dissenting views” and that “students must be allowed to rebel,” stressing that the revolutionization of the cultural realm should unfold as a masses-driven process that encouraged open political contestation and ideological initiative.^28^ For Mao, the maladies afflicting the cultural and educational spheres had reached a breaking point. Determined to force a decisive rupture, he embraced the banner of cultural revolution as a frontal assault on the entrenched cultural and ideological establishment.

In late March, Mao’s antagonism toward the CCP’s ideological apparatus reached a new pitch. This sharpened hostility was provoked by escalating tensions between leaders in Shanghai and Beijing over the handling of the *Hai Rui* affair, following the February Outline that imposed new constraints on radical political criticism. When Zhang Chunqiao and his radical associates in Shanghai were rebuffed by Peng Zhen, Jiang Qing and Kang Sheng framed this as a veiled challenge to Mao’s personal authority. Kang Sheng’s provocative warning—“If we do not launch a cultural revolution now, the old, the middle-aged, and the young alike will all become targets of attack”—stoked Mao’s anger and heightened his sense of urgency (Li, 2015: 60–61). In response, Mao lashed out at the Central Propaganda Department, accusing it of shielding the cultural establishment and suppressing leftist critics such as Yao Wenyuan. He derided it as the “Palace of the King of Hell,” declaring, “We must overthrow the King of Hell and liberate the little devils!” Mao now recast the conflict as a confrontation with the Party Center itself, charging that entrenched interests continued to obstruct ideological transformation in academic and educational institutions. He warned that “we stand on the threshold of a cultural revolution,” insisting that “as the socialist revolution deepens, their resistance intensifies,” and concluding that “this cultural revolution is essential to combating revisionism.”^29^

Mao’s rhetoric escalated still further in a March 30 meeting with Kang Sheng, Zhang Chunqiao, and Jiang Qing. To heighten the stakes, he invoked a pointed allegory that would soon circulate as a powerful idiom of rebellion, “When the Monkey King rebels against the Celestial Palace, do you side with the Monkey King or with the celestial generals and soldiers, with the Celestial Emperor?” Identifying revolutionary youth with the Monkey King, Mao unambiguously aligned himself with the younger generation and entertained the prospect of a radical rupture with the party’s institutional machinery: “We might as well disband the Five-Person Group, the Central Propaganda Department, the Beijing Municipal Party Committee, or, for that matter, any provincial or municipal party committees.” He then pressed a series of questions that exposed his existential anxiety about the party’s future, asking, “Can the cultural revolution be carried through to the end? Can we politically withstand the pull of revisionist tendencies? Will the party’s leadership itself succumb to revisionism?” He then voiced a more personal unease, framing the struggle in generational terms. “We are all getting old,” he admitted. “Cultural revolution is a long-term and arduous task. I cannot complete it within my lifetime, but it must be carried through to the end” (Documentation Research Office, 2013: 5.572–73).

Mao’s pronouncement marked a decisive turning point. On March 31, Kang Sheng returned to Beijing to deliver Mao’s directives: to raise high the banner of the Proletarian Cultural Revolution 无产阶级文化革命, to subject literature, history, and philosophy to thorough ideological criticism, and to expose the anti-party and anti-socialist positions of reactionary academic authorities (Documentation Research Office, 2013: 5.573). Armed with this marching order, the party’s organizational machinery moved swiftly, initiating a coordinated campaign of ideological mobilization targeting academic and cultural institutions across the system and translating Mao’s escalating rhetoric into concrete political intervention.

On April 2, Zhou Enlai wrote to Mao with concrete proposals. He outlined a series of measures, including organizing ideological ranks, intensifying the struggle against reactionary academic authorities, and drafting a new central directive for Mao’s review. Zhou also sharply criticized the February Outline submitted by Peng Zhen’s Five-Person Cultural Revolution Group as fundamentally flawed, recommending that the Central Secretariat either revise it thoroughly or abandon it altogether (Documentation Research Office, 2013: 5.573). From April 9 to 12, Deng Xiaoping chaired meetings of the Central Secretariat to deliberate on the Cultural Revolution’s implementation. These meetings yielded two consequential decisions: first, to issue a formal notice repudiating the Five-Person Group’s report; and second, to create a new body—the Cultural Revolution Document Drafting Group 文化革命文件起草小组—charged with preparing authoritative documents to define and direct the emerging movement (Documentation Research Office, 2009: 3.1907–1908). Taken together, these steps marked not merely a break with Peng Zhen’s managed approach to cultural criticism and reform, but also the leadership’s first coordinated effort to realign the party apparatus toward an ideological offensive decisively shaped by Mao’s increasingly uncompromising position.

Between April 16 and 24, Mao convened another Politburo meeting in Hangzhou. The agenda focused on the “intense criticism of Peng Zhen” and on a series of consequential decisions, including dissolving the Five-Person Group, rescinding its report, and creating a new body to direct the unfolding movement. On April 22, Mao addressed the meeting, situating these measures within a broader ideological struggle. “This is not merely an issue with Wu Han,” he declared, “It is a struggle over ideology, one that extends widely. There are many individuals involved at the central level, in provinces, in major regions, and within departments like cultural bureaus and propaganda ministries.” Mao stressed the need for a phased, systematic struggle, calling for the removal of bourgeois figures at the provincial and municipal levels—at least one or two in each region. He voiced frustration that party leaders had failed to grasp the severity of the situation, attributing their inaction to weak oversight and organizational laxity, a shortcoming he acknowledged extended even to himself. Concluding his remarks, Mao articulated what he presented as the core principle of revolutionary transformation: “Without destruction, there can be no construction. Thorough destruction will bring about construction; within destruction lies construction” (Documentation Research Office, 2013: 5.580). By casting destruction as both necessary and generative, Mao underscored his conviction that dismantling existing structures was essential for forging a new social and political order. This rationale would soon become central to the movement that followed.

On April 23, in the course of reviewing a report from the Ministry of Higher Education, Mao issued a directive declaring that “university faculty and students in all regions, as well as middle school teachers and students, must all participate in the cultural revolution movement 都应参加到文化革命运动中去” (Documentation Research Office, 1998a: 46–47). Later that day, Deng Xiaoping proposed that the movement be concluded within six months, a timetable to which Mao assented (Party History Research Office of the Zhejiang Provincial Committee of the Chinese Communist Party, 1993: 267). This initial time frame points to a campaign envisioned as bounded and time-limited, one focused primarily on reordering the cultural and ideological spheres rather than unleashing open-ended political upheaval. What followed, however, quickly exceeded these initial parameters: a campaign conceived as a targeted intervention soon spiraled beyond its original scope, destabilizing the party-state in ways that ultimately outpaced Mao’s own expectations.

The two Politburo meetings held in Hangzhou in the spring of 1966 marked a watershed moment in the history of the PRC. It was here that Mao’s resolve to abandon the measured, institutionally managed approach to cultural rectification associated with Peng Zhen and the party’s propaganda apparatus fully crystallized, giving way to a far more militant course. According to Qiu Huizuo, then director of the General Logistics Department of the PLA, General Yang Chengwu, acting chief of staff of the PLA, briefed senior military officers on the April meeting. Yang delivered an unambiguous message: “A counteroffensive is imminent.” He elaborated that the “counteroffensive means Chairman Mao has resolved to push back at the cultural revolution they are directing 要反击他们正在领导进行的文化革命,” thereby explicitly identifying Peng Zhen and his associates—the architects of the party’s earlier cultural policy—as the principal targets. Yang further relayed Mao’s blunt declaration: “Our cultural revolution is about to begin” (Qiu, 2011: 432–33).

## The May 16 Notice in Cultural-Revolutionary Context

Between May 4 and 26, 1966, Liu Shaoqi chaired an expanded Politburo meeting in Beijing that officially launched the militant cultural campaign. General Liao Hansheng, political commissar of the Beijing Military Region and a participant in the meeting, later recalled that Liu opened the proceedings by announcing that the meeting would primarily address the “two-line struggle” associated with the cultural revolution (Liao, 2003: 239). During the sessions, Kang Sheng delivered a sweeping indictment of Peng Zhen, charging him with “anti-Party and anti-Mao” offenses. He further presented a series of recent directives from Mao that sharply criticized Peng Zhen, Lu Dingyi, and the broader cultural and propaganda apparatus. Zhang Chunqiao situated Yao Wenyuan’s criticism of Wu Han within a wider ideological confrontation, casting it as a pivotal episode in the escalating cultural struggle (Bu, 2008: 74–79). In his remarks, Liu Shaoqi stressed the need to study Mao Zedong Thought for guiding the militant cultural campaign. Striking a tone of self-criticism, he acknowledged earlier failures: “On this issue, we were muddle-headed; we neither fully understood these critical matters nor took them seriously. Consequently, our work was ineffective, myself included” (Documentation Research Office, 1996c: 638).

On May 16, 1966, the Politburo adopted a pivotal directive that would later be known as the May 16 Notice (hereafter, the Notice). It was drafted by the recently formed Cultural Revolution Document Drafting Group—the precursor to the CCRG—composed primarily of propaganda officials and cultural radicals, including Jiang Qing, Kang Sheng, Chen Boda, Zhang Chunqiao, Yao Wenyuan, Wang Li, among others. Substantially revised by Mao himself, the Notice became the foundational text of the burgeoning movement. A year later, *People’s Daily* celebrated it as a “great historical document” that articulated the “theories, principles, and policies of the Great Proletarian Cultural Revolution” and “sounded the clarion call” for the movement (Renmin ribao, May 18, 1967).

Yet the content of the May 16 Notice itself points to a more circumscribed conception of “revolution” at the time than later official formulations and scholarly interpretations would suggest. Like Jiang Qing’s Summary, the Notice offered a sweeping indictment of China’s cultural and ideological spheres, declaring that “for many years, our newspapers, broadcasts, journals, books, textbooks, literary and artistic works, films, theater, folk art, fine arts, music, dance, and so forth have been inundated by demons and monsters, as well as reactionary bourgeois authorities.” On this basis, the Notice proclaimed the advent of a “great surge of a Proletarian Cultural Revolution,” charged with dismantling the “decadent ideological and cultural strongholds” of bourgeois and feudal influence. Its language intensified an already circulating sense of cultural crisis, casting the newly initiated movement as a decisive effort to uproot entrenched bourgeois authorities within the cultural and ideological spheres.

Throughout Mao’s final decade, China’s official media consistently identified the May 16 Notice as the programmatic document that inaugurated the Great Proletarian Cultural Revolution. Within this canonical interpretation, Mao initiated the movement to combat what he perceived as the infiltration of bourgeois elements into the party. Only through mass mobilization—conceived as an open-ended, bottom-up struggle—could this usurped power be reclaimed. These premises were subsequently distilled into what came to be known as the “theory of continuing revolution,” with the Notice retrospectively elevated as its authoritative statement. This reading outlived Mao’s lifetime, shaping official narratives and scholarly and popular understandings for decades thereafter.

Yet a fundamental question remains insufficiently specified: what, precisely, was the “Cultural Revolution” envisioned in the May 16 Notice? This article argues that the historical record does not sustain this long-dominant interpretation. A close rereading of the Notice suggests that it was decidedly not a revolutionary summons to the masses to rise against the party-state and its bureaucratic apparatus. Instead, it functioned as an internal party directive, oriented toward a far more circumscribed set of concerns, whose political implications would only later be expanded—and transformed—through subsequent developments.

First, the May 16 Notice did not seek to initiate a “great revolution” through “open, comprehensive, and bottom-up mobilization.” Its immediate purpose was far more limited: to repudiate the earlier February Outline, which had endorsed a managed and procedurally restrained approach to cultural revolution. Denouncing the February Outline as “fundamentally erroneous” and in direct violation of Mao’s instructions, the Notice called for the dissolution of the Five-Person Group and the establishment of a new body to mount a counteroffensive in the cultural and ideological spheres. Second, the Notice was a classified internal document, circulated only among party cadres above a designated rank; it was not intended for public release at the time of its adoption (MacFarquhar and Schoenhals, 2006: 41). Although its core messages quickly spread and became widely known through various channels, thereby catalyzing broader mobilization, the full text was not formally published until a year later. Finally, if the Cultural Revolution is understood as having originated as a radical cultural movement, then its first systematic articulation did not appear in the May 16 Notice at all. That role was instead played by the Summary, drafted under Jiang Qing’s stewardship. As discussed earlier, it was the Summary—not the Notice—that offered the earliest programmatic vision of a militant cultural revolution, spelling out its aims, targets, and justificatory logic.

Toward the conclusion of the May 16 Notice, Mao personally inserted a sharply worded passage that would later assume particular weight in the document’s interpretation. He warned of “bourgeois representatives who have infiltrated the party, government, military, and various cultural sectors,” portraying them as counterrevolutionaries poised to “seize power and transform the dictatorship of the proletariat into a dictatorship of the bourgeoisie.” He further cautioned that some of these figures, though not yet exposed, were “trusted and being groomed as successors to us.” By drawing a direct parallel to Nikita Khrushchev—his archetypal figure of revisionism—Mao warned that such figures were “lying beside us” (Documentation Research Office, 2013: 5.579), thus stressing the proximity of the danger and the need for vigilance within the party. As MacFarquhar and Schoenhals have argued, this key passage—added by Mao himself—signaled that the Notice pointed toward a political future Mao had already prescribed (MacFarquhar and Schoenhals, 2006: 47).

These politically charged epithets—especially references to “trusted successors” and “Khrushchev-like figures lying beside us”—were subsequently read as clues to Mao’s intended targets. Scholars who argue that Mao launched the Cultural Revolution with the specific aim of eliminating Liu therefore read the Notice as a coded indictment of his principal rival. From this vantage point, Mao—fully cognizant of Liu’s entrenched institutional power—appears as a strategist biding his time, waiting for conditions to ripen before striking. Chinese historian Bu Weihua exemplifies this line of reasoning, contending that while Peng Zhen appeared to be the immediate target, “Mao’s real aim was the central leadership headed by Liu Shaoqi. When conditions were not yet ripe, Mao used phrases like ‘figures like Khrushchev’ as a veiled reference” (Bu, 2008: 81). This narrative has gained wide currency in both scholarly and popular accounts. Yet this reading rests on a retrospective alignment between Mao’s language and later outcomes, leaving unresolved a more immediate problem: how the May 16 Notice functioned politically at the moment of its issuance, before its language hardened into a determinate script.

Read against the immediate political landscape of 1965–1966, however, this interpretation relies on a retrospective logic, projecting causal relationships derived from later developments rather than engaging the circumstances in which the May 16 Notice was produced. A closer examination of the document in its contemporaneous setting suggests that references to “Khrushchev-like figures” were more plausibly directed at Peng Zhen and key figures within the party’s propaganda and cultural apparatus.

As a member of the Politburo and Beijing’s party chief, Peng Zhen exercised authority over the administration and security of the national capital—a power that extended well beyond municipal governance. He also served as vice chairman of the National People’s Congress and as the second-ranking figure in the CCP’s Central Secretariat, outranked only by Deng Xiaoping, and played a central role in legal affairs, foreign policy, and party administration. As head of the Cultural Revolution Five-Person Group, Peng oversaw ideological and cultural work at the national level—the very domains Mao sought to dismantle and remake. Peng’s prominence was publicly affirmed in a *People’s Daily* report on September 13, 1964, which listed him immediately after Liu Shaoqi, Zhou Enlai, and Deng Xiaoping and identified him as one of Mao’s “closest comrades-in-arms” 最亲密的战友 (Renmin ribao, September 13, 1964).^30^ Against this backdrop, rumors circulated among Beijing cadres that Peng had become Mao’s successor (Nie, 2005: 113). Peng’s standing was likewise noted in a CIA analysis from August 1965, which identified him as a leading contender in a post-Mao succession scenario, speculating that in the event of Liu’s premature exit, both Peng Zhen and Deng Xiaoping “seem the strongest candidates for the top position” (Central Intelligence Agency, 1965: 4). Seen from the political configuration of the mid-1960s, reading the May 16 Notice as a coded indictment of Liu Shaoqi depends less on available evidence than on hindsight, in a political landscape where Peng Zhen was the more immediate and structurally plausible referent.

There is no compelling evidence that Peng Zhen’s political demise in the spring of 1966 resulted from his affiliation with an alleged Liuist faction. Instead, his fall arose from a more immediate conjuncture: his obstinate handling of the *Hai Rui* affair and Mao’s mounting dissatisfaction with the party’s cultural and ideological work under Peng’s stewardship. As this article argues, the May 16 Notice marked the opening of a militant ideological and cultural campaign. The available record does not support claims that Mao’s warnings in the Notice constituted a veiled attack on Liu Shaoqi, nor does it suggest that Mao had, at this stage, envisaged an unrestrained mass movement directed against the party-state and its top leadership. Although phrases such as “bourgeois representatives infiltrating the party” construed internal danger in stark terms, they stopped short of authorizing autonomous mass action. This distinction is critical: while the ideological conditions for subsequent radicalization were being assembled, the call for mass participation as an independent political force had not yet been issued—a point to which this article will return.

At the core of the May 16 Notice was an outright repudiation of Peng Zhen and the February Outline. Mentioned by name 94 times, Peng was singled out as the chief obstructionist to Mao’s vision of cultural change. The Notice accused the February Outline of downplaying class struggle, reducing ideological conflict to mere academic disputes, shielding bourgeois intellectuals, suppressing leftist critics, and stalling the advance of a “proletarian cultural revolution.” To reinforce its charges, the Notice appended a lengthy chronology of “Major Events in the Struggle between Two Lines on the Cultural Front,” which detailed Peng Zhen’s alleged offenses.

Wang Li, a key figure in drafting the Notice and later an influential CCRG member, recalled Jiang Qing’s key role in the drafting process. Her approach closely mirrored the methods she had employed at the PLA symposium two months earlier, where she organized daily film screenings to subject ideologically problematic works to sustained scrutiny. Applying similar strategies to the formulation of the Notice, Jiang Qing pressed for the systematic purging of cultural products she deemed ideologically flawed. Her active engagement was driven in no small part by longstanding grievances against the Central Propaganda Department and the Beijing party leadership under Peng Zhen, particularly their resistance to her controversial push to reform Peking Opera. Throughout the drafting process, she directed her criticisms squarely at Peng and the propaganda establishment, lamenting, “In Beijing, I have been completely isolated and ignored—‘not even a needle can be inserted, not a drop of water can penetrate.’” Recounting her repeated but unsuccessful efforts to secure institutional backing, she explained that this isolation compelled her to seek allies elsewhere, turning to Shanghai and increasingly relying on the military. To facilitate the drafting, she supplied the minutes of the PLA symposium, offering them as a model for cultural militancy (Wang, 2008: 382–83).

The Notice’s reference to “those in power within the party taking the capitalist road” is frequently invoked as evidence that Mao intended to target senior leaders, above all Liu Shaoqi. Yet such readings remain speculative and rest on thin evidentiary foundations. A key source of misinterpretation lies in the frequent conflation of the May 16 Notice with the “Decision of the Central Committee of the Chinese Communist Party on the Great Proletarian Cultural Revolution” (commonly known as the “Sixteen Articles”) issued in August. This slippage collapses two texts produced under markedly different political conditions, obscuring their distinct purposes and scopes. A careful comparison makes this divergence clear. The Sixteen Articles—a later document—explicitly named “capitalist roaders within the party” as the principal target, thereby authorizing a broad political offensive. By contrast, the May 16 Notice employed more restrained language, referring to “those in power within the party taking the capitalist road” only once and in a qualified manner, specifying officials who “support and protect bourgeois academic authorities.” This asymmetry is analytically significant: It highlights the Notice’s relatively limited agenda, clearly distinguishing it from the openly political and expansive thrust of the Sixteen Articles.

The name “Khrushchev” entered Chinese political discourse in the mid-1960s as a shorthand for ideological betrayal. While its usage in the May 16 Notice has often been interpreted as a veiled attack on Liu Shaoqi, the evidentiary basis for such a reading remains thin and inconclusive. Although tensions between Mao and Liu had clearly intensified since the early 1960s—stemming from disagreements over the legacy of the Great Leap Forward in 1962, the course of economic recovery, and the handling of the Socialist Education Movement in 1964—these conflicts alone do not warrant the conclusion that Mao launched the Cultural Revolution with a preconceived plan to remove Liu. Instead, Liu’s downfall may be better understood as a contingent outcome of the movement’s escalating radicalism rather than its initial aim.^31^ Disentangling the indeterminate alignments of the early intra-party struggle from the hardened accusations that emerged from the Cultural Revolution is therefore essential for a more accurate understanding of Liu’s fate.^32^

The May 16 Notice marked the official launch of the militant Cultural Revolution, conceived as an effort to purify China’s cultural and ideological landscape. Its principal targets included “bourgeois academic authorities,” as well as “black gangs”—a stigmatizing label applied to groups accused of ideological subversion—and the so-called “royalist” officials charged with protecting them. On May 31, 1966, with Mao’s approval, Chen Boda, head of the newly established CCRG, took over control of *People’s Daily* (Documentation Research Office, 2013: 5.588–89). Upon taking charge, Chen defined the paper’s mission in unmistakably militant terms: to eradicate bourgeois ideology, reassert command over public discourse, and eliminate all remnants of “old thought, old culture, old customs, and old habits.” To accelerate momentum, Chen ordered the publication of a rousing editorial. The initial draft—entitled “Continue the Struggle, Push the Great Proletarian Cultural Revolution to the End”—was judged insufficiently combative. Seeking greater ideological intensity, Chen reworked the title as “A Cultural Revolution that Sweeps Away All the Demons and Monsters,” before condensing it further into the more incendiary “Sweep Away All the Demons and Monsters” (hereafter “Sweep Away”). Published on June 1 as the front-page editorial of *People’s Daily* and rapidly reprinted nationwide, the piece helped set the ideological tone for the unfolding militant cultural campaign.^33^

For the first time, the “Sweep Away” editorial publicly disclosed key elements of the May 16 Notice. Declaring that “a new peak of the proletarian cultural revolution is rising,” the editorial cast the unfolding movement as an existential struggle over ideology and culture, stretching from the founding of the PRC to the present moment (Renmin ribao, June 1, 1966). Crucially, the phrase “demons and monsters” was not Chen Boda’s invention, but a familiar component of the CCP’s rhetorical repertoire. In 1966, it initially targeted “bourgeois academic authorities” and “rightists.” As the militant cultural campaign gathered momentum, however, its referent widened, coming to encompass “capitalist roaders” within the party itself.

The “Sweep Away” editorial, published on June 1, 1966, quickly became a rallying cry for the emerging Cultural Revolution. The following day, *People’s Daily* ran another major editorial, “A Great Revolution That Touches People’s Souls,” which presented ideological and superstructural battles as an existential necessity for socialism. Rejecting the notion that cultural and intellectual conflicts were secondary, the editorial insisted that they were central to preventing capitalist restoration. This line of argument was amplified on June 5, when the *PLA Daily* published a 13,000-character article, “Hold High the Great Banner of Mao Zedong Thought and Carry the Great Proletarian Cultural Revolution Through to the End.” Synthesizing themes from Jiang Qing’s Summary, the May 16 Notice, and the “Sweep Away” editorial, the article accused bourgeois figures in the cultural sphere of persistently opposing Mao’s revolutionary vision by disseminating pernicious ideas through newspapers, radio broadcasts, textbooks, literature, and performing arts. On June 11, *Red Flag* pushed the rhetoric still further with an editorial entitled “Long Live the Great Proletarian Cultural Revolution,” invoking the Soviet Union as a cautionary case. It attributed the rise of revisionism after Stalin’s death, along with events such as the 1956 Hungarian uprising, to the Soviet leadership’s failure to carry out a thorough cultural revolution. State power, it argued, could not be secured by the “barrel of the gun” alone; the “pen,” as an instrument of ideological struggle, was equally indispensable. Without sustained ideological and cultural combat, the editorial warned, the revolutionary state itself would be imperiled (Hongqi, June 11, 1966).

The cultural offensive intensified further in the weeks that followed. On July 1, *Red Flag* reprinted Mao’s 1942 “Talks at the Yan’an Forum on Literature and Art,” a foundational text that had long structured the CCP’s approach to cultural work. Alongside Mao’s 1940 essay “On New Democracy,” which contained his earliest systematic articulation of the idea of cultural revolution,^34^ the Yan’an Talks were reintroduced not merely as historical documents but as doctrinal touchstones and operational guides for the unfolding movement. This return to Mao’s pre-1949 writings signaled a deliberate recentering of the Cultural Revolution’s ideological genealogy, reaffirming the primacy of cultural transformation and underscoring the belief that continuous revolution required the relentless remaking of China’s cultural landscape.

## “Essentially a Great Political Revolution”

A sweeping ideological and cultural offensive took shape in late spring and early summer of 1966, aimed at purging literature, the arts, and intellectual discourse of perceived bourgeois influences. Scholars, writers, and artists were publicly denounced as politically suspect, while teachers faced accusations of corrupting students—especially those from working-class backgrounds. School and propaganda officials came under scrutiny for ideological laxity and failure to uphold party doctrines (Bu, 2008: 130–40). These attacks and purges marked the launch of what was officially designated as the Cultural Revolution—a term that, for many contemporaries, carried its most literal meaning: a revolutionary assault on China’s ideological terrain and an effort to remake its cultural, literary, artistic, and educational institutions.

Notably, Mao himself seems to have understood the emerging movement in precisely these terms. In early June 1966, the CCP’s top leadership assembled in Hangzhou to deliberate on the Cultural Revolution. During the June 10 meeting, Mao articulated his objectives in a conversation with Hồ Chí Minh, offering one of the clearest contemporaneous indications of how he understood the campaign at this early juncture:

Mao: Our current struggle began last November and has continued for more than seven months. It was initially sparked by Yao Wenyuan, a young man, who raised questions about the upright officials 清官. Since then, we have moved beyond the narrow debate over “upright” versus “corrupt” officials and embarked on the broader Cultural Revolution. This campaign targets key ideological and institutional domains, including education, literature, the arts, academia, philosophy, history, publishing, and journalism. The literary and artistic fields alone encompass multiple subfields—drama, film, music, fine arts, and sculpture. Within theater, for example, there are Peking Opera and hundreds of regional styles. At present, *dazibao* [big-character posters] are the primary method. While newspapers are full of activity, it is *dazibao* that have generated the greatest momentum and mobilized the broadest participation.

Hồ: I recall seeing such posters during my visit to China in 1957.

Mao: This time, the scale and depth of the movement far surpass those of 1957. It may lead to the downfall of hundreds, perhaps several thousands, particularly in academia, education, journalism, publishing, and the arts, as well as in universities, middle schools, and primary schools. Previously, we lacked our own trained personnel and had to rely on those teachers from the Kuomintang. Many of those we absorbed—educators, journalists, actors, novelists, painters, and filmmakers—have now infiltrated our party. Now, you can understand why this Cultural Revolution is necessary.

Hồ: This movement has been deeply enlightening for me. Before coming to China, I was unaware of the intensity and scope of the struggle. Your words are far clearer than the reports I read. The situations China faces also exist in Vietnam. What China pursues, Vietnam must also follow—perhaps on a smaller scale. Our conditions are fundamentally similar.

Mao: Indeed, they are likely quite similar. You, too, have primary, secondary, and university teachers, many of them old intellectuals.^35^

This exchange is particularly revealing, as it distills Mao’s early understanding of the Cultural Revolution as a campaign to remake China’s cultural and educational institutions. Recorded before the onset of mass insurgency and the consolidation of later retrospective narratives, it offers a rare, relatively unmediated glimpse into how Mao initially conceived the movement, before its escalation in largely unanticipated ways.

That same day, Mao addressed the Politburo, stressing the need for an unrestricted and uncompromising approach to the Cultural Revolution. Framing it as a masses-driven campaign to expose and criticize ideologically suspect elements, he insisted that no one—irrespective of rank, seniority, or reputation—should be exempt from scrutiny. “In the Cultural Revolution, we must act boldly, without fear of disorder, and mobilize the masses fully,” he declared. “This movement must be carried out on a large scale, so that all reactionary elements can be exposed.” Only through such audacity, he argued, could the cultural sphere be wrestled from entrenched authority. He also praised the emerging cohort of young activists, identifying them as indispensable to the movement: “We must rely on these individuals to see the Cultural Revolution through to its conclusion” (Documentation Research Office, 2003: 2.1417).

On June 12, during discussions on the movement’s implementation, Mao further elaborated on its central objectives, proposing that educational reform be at its core. He outlined a phased strategy, beginning with an assault on the establishment—what he described as the “seizure of power”—followed by a sweeping transformation of pedagogy, curricula, and the broader educational system. Among his proposed structural changes was a hybrid model of university admission, combining high school recommendations with competitive exams. Mao also called for postponing university entrance exams by six months to accommodate the demands of the Cultural Revolution. Besides admissions, he advocated a comprehensive overhaul of curricular materials, removing content he viewed as ideologically harmful. Championing a revolutionary model of education, he promoted mutual teaching and learning among professors, teachers, and students. This emphasis on collective, ideologically driven learning reflected Mao’s broader understanding of the Cultural Revolution as a means of transforming everyday educational practice (Documentation Research Office, 2013: 5.593). The next day, on June 13, the Party Center issued a directive formally postponing that year’s university admissions process, including the national entrance examinations, justifying the decision as necessary for the thorough implementation of the Cultural Revolution (Renmin ribao, June 18, 1966).

Later that month, while visiting Albania, Zhou Enlai addressed a mass rally in Tirana, characterizing the movement as an ideological struggle to prevent the “restoration of capitalism.” He proclaimed: “In recent months, we have launched a vigorous cultural revolution, which is fundamentally a sharp class struggle in the realm of ideology. At its core, it is a battle between restoration and anti-restoration.” His remarks closely tracked Mao’s language, reinforcing the movement’s ideological and cultural focus (Documentation Research Office, 1997: 3.38). Albanian records provide further detail on Zhou’s discussions with host leaders, in which he presented the Cultural Revolution as a long-prepared initiative centered on the ideological sphere, describing it as an upheaval that “deeply shakes people’s souls.”^36^

By mid- to late June 1966, statements from Mao and other senior CCP leaders suggest that they understood the unfolding Cultural Revolution as a militant offensive aimed at reshaping the intellectual and cultural foundations of Chinese society in accordance with radical revolutionary principles. Yet within a matter of weeks a marked shift became apparent in both the leadership’s understanding of the movement and its official rhetoric. What had begun as an ideological offensive against bourgeois intellectuals and cultural authorities rapidly expanded into a sweeping political assault, with its objectives and impact substantially redefined. This reorientation is best illustrated through a comparative analysis of two pivotal texts produced between May and August 1966: the May 16 Notice and the Sixteen Articles, which together mark critical turning points in the movement’s changing priorities and scope.

Issued in mid-May 1966, the May 16 Notice established the ideological scope for what was initially a militant cultural initiative. By August, however, the campaign had undergone a rapid expansion, with its objectives substantially reconfigured. The adoption of the Sixteen Articles signaled a transition from cultural purges to far more open-ended and mass-based political mobilization.^37^ While both texts played key roles in shaping the Cultural Revolution, their divergence highlights the movement’s transformation from targeting cultural elites to mounting a broader political challenge to entrenched structures of party-state authority.

The Sixteen Articles reaffirmed the May 16 Notice’s call for a radical assault on and transformation of culture and ideology, proclaiming at its very beginning: “Although the bourgeoisie has been overthrown, they still attempt to use the old ideology, culture, customs, and habits of the exploiting classes to corrupt the masses, win over hearts, and restore capitalism. In contrast, the proletariat must resolutely counter all ideological challenges posed by the bourgeoisie and use its own new ideology, culture, customs, and habits to transform the ideational landscape of the entire society.”^38^ While this rhetoric of the “four olds” and “four news” echoed the May 16 Notice and Chen Boda’s “Sweep Away” editorial, the Sixteen Articles went further in redefining both the movement’s targets and its operational logic. Yet despite their critical differences, scholarly discourse has frequently collapsed the two texts into a single, continuous script, thereby flattening the shifting political contours that marked the Cultural Revolution’s early months.

The Sixteen Articles and the May 16 Notice diverge in three key respects: the movement’s objectives, their treatment of bureaucratic resistance, and their respective visions of mass mobilization. First, the Sixteen Articles redefined the Cultural Revolution’s aims and principal targets. The most striking shift was the campaign’s reorientation from attacking “bourgeois academic authorities” and “demons and monsters” to a direct assault on “those in power within the party who take the capitalist road.” Commonly abbreviated as “capitalist roaders in the party,” these figures—party officials accused of obstructing socialist transformation—became the central target of revolutionary struggle. Article 1 outlined three primary objectives: (1) overthrowing party officials allegedly taking the capitalist road; (2) criticizing bourgeois academic authorities and bourgeois ideology; and (3) transforming education, culture, and the superstructure. While cultural and educational transformation remained important, the Sixteen Articles clearly prioritized purging party organizations, marking a shift from combating cultural subversion to directly challenging party authority and hierarchy.

Second, the Sixteen Articles identified bureaucratic resistance within the party and specified mechanisms to neutralize it. Article 2 explicitly warned that “those in power within the party taking the capitalist road” would attempt to suppress the mass movement. At the same time, Article 3 insisted that their removal was a prerequisite for advancing the Cultural Revolution. Although the May 16 Notice had also criticized “bourgeois representatives in the party,” its primary concern was officials who shielded bourgeois intellectuals. By contrast, the Sixteen Articles widened the field of suspicion, implicating a broad range of party officials deemed ideologically unreliable.

Third, the Sixteen Articles introduced a fundamental reorientation of method and strategy. It celebrated the emergence of “previously unknown revolutionary youths” and affirmed their use of *dazibao* and public debates as instruments of “free and open expression of views and bold and fierce criticism.” It further urged party leaders to “trust the masses, rely on the masses, and respect their spirit of innovation,” casting the Cultural Revolution as a masses-driven process from below. By moving beyond top-down initiatives and sanctioning grassroots political agency, the Sixteen Articles conferred legitimacy on mass mobilization and authorized direct challenges to party authority.

A particularly audacious element of this vision drew inspiration from the Paris Commune of 1871, invoking its model of direct popular engagement through collective self-organization and action. The Sixteen Articles accordingly called for the creation of new forms of power through popular elections, public criticism, and the recall of officials. This move marked a clear ideological departure, associating the Cultural Revolution with the idea of a “great revolution” 大革命—one that, as Mao would later insist, required “mobilizing the masses openly, comprehensively, and from the bottom up to expose the dark aspects of our society and the party.”^39^ By legitimating mass movements that contested the party-state bureaucracy, embodied in the distinctly Maoist ideal of “great democracy” 大民主, the Sixteen Articles codified what would soon emerge as the movement’s defining feature: bottom-up rebellion against entrenched party authority. As Wang Li, a key CCRG member involved in drafting the document, later reflected, the movement had in effect turned into “a revolution against the Communist Party itself” 革命革到自己头上 (Wang, 2008: 421).

Following the adoption of the Sixteen Articles on August 8, Lin Biao’s speech at a central conference on August 13 spelled out the movement’s new orientation with striking bluntness. No longer construed as a campaign confined to the ideological and cultural sectors, the movement was now presented as a sweeping purge and reorganization of the ruling Communist Party itself. Lin encapsulated this reorientation in the concepts of the “Three Comprehensives” 三个全面 and the “Three Dismissals” 三个罢官. The former prescribed a systematic approach to cadre assessment—“comprehensive evaluation, comprehensive ranking, and comprehensive reorganization” 全面考察, 全面排队, 全面调整—while the latter mandated the removal of officials who failed to uphold Mao Zedong Thought, obstructed political and ideological work, or demonstrated insufficient revolutionary zeal. Lin summarized these principles into a succinct formula: “In the Cultural Revolution, we must dismiss some officials, promote others, and retain yet some,” noting that this sweeping restructuring of the party carried Mao’s approval (Revolutionary Committee of Central China Normal University, 1968: 321). He reiterated this during a Politburo meeting on December 3, 1966, characterizing the Cultural Revolution as a process of “extensive criticism, scrutiny, and education” 大批判, 大审查, 大教育 applied to the entire party and its cadredom. At its core, he maintained, the movement was a systematic campaign of ideological and organizational purification, aimed at reshaping party leadership in line with Mao’s revolutionary line (Revolutionary Committee of Central China Normal University, 1968: 342).

A closely related reinterpretation was offered by Tao Zhu, newly elevated to the fourth-ranking position in the Politburo. In a speech on August 23, 1966, Tao framed the Cultural Revolution as a test directed at veteran party cadres. “Veteran revolutionaries contributed to the revolution in the past,” he acknowledged, “but the new challenge is to pass the test of socialism. The Cultural Revolution is a critical test, and some of them may fail to pass it.” He emphasized the campaign’s radical permissiveness: “Now it is permissible to expose anything and anyone—no exceptions. Everyone should do their utmost to expose. . . . We cannot doubt Chairman Mao, nor the leadership of the party. Beyond that, entire sectors, grassroots party leaders, or ministry party leaderships may be subject to doubt” (Editorial Office of Guizhou Workers, Guizhou Provincial Workers’ Revolutionary Rebel Command Headquarters, 1967: 165–66). Yet Tao’s attempt to delimit the scope of popular radicalism by insulating the party’s top leadership proved untenable. Even as he spoke, the party bureaucracy and its officials were already under intensifying mass assault, as the movement rapidly exceeded the boundaries he sought to impose.

During this period, Mao’s understanding of the Cultural Revolution appears to have shifted as well. In August and September of 1966, he reportedly described the movement as a “large-scale drill” to prevent and combat revisionism, a political crucible in which “all cadres must be put through the fire” and be compelled to “put up a performance” 表演一番 that would reveal their true political colors (Wang, 1993: 33). This language marked a clear departure from his earlier, more circumscribed conception of the campaign. Following the promulgation of the Sixteen Articles in early August, the movement’s original emphasis on “cultural revolution” was progressively attenuated, as political mobilization against party-state authority came increasingly to the fore. This reorientation was crystallized on September 15, when Mao, while revising Lin Biao’s speech for a Red Guard rally, inserted a key passage redefining the movement’s purpose: “The fundamental contradiction the Great Proletarian Cultural Revolution seeks to address is the contradiction between the proletariat and the bourgeoisie, between socialism and capitalism. The focus of this movement is to combat those in power within the party who take the capitalist road” (Documentation Research Office, 1998a: 135). Notably, this revision dispensed with the earlier emphasis on revolutionary cultural struggle, which had figured centrally in the May 16 Notice and other official pronouncements only months earlier. The Cultural Revolution was thus reframed in explicitly political terms, with an unmistakable focus on identifying, testing, and eliminating alleged enemies within the party itself.

This shifting political narrative crystallized on December 26, 1966, during Mao’s birthday dinner, attended by Jiang Qing, Chen Boda, Zhang Chunqiao, Wang Li, and Yao Wenyuan. On that occasion, Mao offered the following blunt diagnosis of the movement’s targets: “The issue lies within the party; fortresses are most easily breached from within. . . . The Cultural Revolution represents a full-scale assault on the bourgeois and petty bourgeois elements within the party.” The dinner concluded with a final toast from Mao that distilled the movement’s mounting momentum and expanding scope: “To comprehensive nationwide class struggle!” (Wang, 2008: 202–23).

Mao’s redefinition of the Cultural Revolution was quickly translated into an official public line. The joint New Year editorial in *Red Flag* and *People’s Daily* soon articulated Mao’s latest directive in equally unambiguous terms: “Nineteen sixty-seven will be a year of comprehensive, nationwide class struggle.” Moving beyond schools, cultural institutions, and government offices, the editorial called for extending the Cultural Revolution into factories and rural villages, signaling a significant expansion of the movement’s terrain. The ultimate objective, it claimed, was to ensure that “Mao Zedong Thought dominates all fronts” (Renmin ribao, January 1, 1967). Reflecting on this trajectory, Lin Biao later characterized it as “essentially a great political revolution but achieved through a cultural revolution,”^40^ a formulation that captured how far the campaign had moved from the cultural and ideological initiatives that had marked its early phase.

## Concluding Remarks: Beyond Whig History

Between late spring and early fall of 1966, the Cultural Revolution moved across distinct, only partially articulated political arenas, whose scope and boundaries remained unstable in practice. What initially took shape as an assault on “bourgeois intellectuals,” “reactionary authorities,” and various cultural “demons and monsters” was reconstituted, within months, as a “great political revolution” directed against putative enemies inside the Communist Party. Yet the ruptures and redefinitions that marked this consequential discontinuity have received surprisingly limited sustained scrutiny in existing scholarship.

This article advances two closely related arguments that help reassess both the origins of the Cultural Revolution and its early dynamics. First, Mao’s decision to initiate the Cultural Revolution is best understood as the culmination of a long-developing revolutionary cultural project, rather than as the opening move of a premeditated mass political purge. In the wake of the Great Leap Forward, Mao became increasingly dissatisfied with developments in literature, the arts, and education, which he interpreted as symptoms of ideological dilution and bureaucratic retrenchment. The militant cultural offensive launched in 1966 thus reflected Mao’s effort to reassert ideological struggle and remake cultural authority, but not yet to unleash a general assault on party power. Seen in this light, the early Cultural Revolution takes shape as a culturally driven initiative with its own logic, one that cannot be reduced to tactical maneuvering or read retroactively through the outcomes it later produced.

Second, the militant cultural movement that crystallized in the May 16 Notice differed sharply in character and scope from the mass political insurgency that followed. The rupture between these two configurations did not mark a shift from culture to politics, but a transformation in the structure and authorization of political action. While the initial mobilization remained largely bureaucratically orchestrated and driven by cultural militancy, the subsequent episode—formalized in the Sixteen Articles—marked a pronounced shift as the movement expanded into direct challenges to party authority. Roderick MacFarquhar’s dramaturgical vocabulary may be helpful here: the unfolding of the Cultural Revolution can be understood as two analytically distinct “acts.” Mao unmistakably orchestrated the first—a cultural purge—yet the second, marked by political escalation, did not simply extend from his initial aims. It took shape through the interaction of political maneuvering, interpretive escalation, and unintended consequences that exceeded any single actor’s control, underscoring the limits of intentionalist and teleological accounts of its early trajectory.

Taken together, these dynamics also call into question the common assumption that Mao launched the Cultural Revolution as the execution of a carefully preconceived political design. The predominance of this thesis in the historiography rests largely on retrospective conjecture about Mao’s intentions, a mode of explanation whose evidentiary foundations remain limited when applied to the movement’s early course.

This article seeks to reconsider conventional historical narratives by arguing that the Cultural Revolution, in its formative phase between early and late 1966, unfolded in discrete and discontinuous episodes. If a rupture indeed separated the early “cultural revolution” from the later “political revolution,” why has it escaped the attention of both contemporary observers and later scholars who have studied this period? The persistence of this historiographical blind spot warrants an inquiry into the entrenched interpretive frameworks and methodological constraints that have obscured this critical discontinuity—an inquiry that must reckon with the extent to which later developments in the Cultural Revolution have shaped understandings of its initial phase.

I suggest that this oversight may stem in large part from the movement’s own unfolding trajectory. Prevailing narratives tend to anchor their analyses in the dramatic developments following August 1966: the issuance of the Sixteen Articles, the rise of the Red Guard movement, Mao’s call to “bombard the headquarters,” the downfall of Liu Shaoqi, and the eruption of mass rebellions. These moments—marking the movement’s expansion and intensification—have come to define how earlier events are understood. As a result, episodes such as the *Hai Rui* affair are often dismissed as a mere prelude to the “great political revolution.” At the same time, the cultural struggles of the early phase are reduced to calculated maneuvers dictated by Mao’s political strategy. This retrospective framing obscures the early Cultural Revolution and renders “cultural revolution” structurally invisible. A similar pattern is evident in the treatment of foundational texts from this period. The May 16 Notice, which signaled the beginning of a cultural counteroffensive, has been subsumed within the interpretive framework of the later Sixteen Articles, thus diminishing its distinctive textual logic and political function. Such conflations exemplify what Herbert Butterfield famously criticized as the Whig interpretation of history: an anachronistic mode of reasoning that reads early events primarily through the lens of their presumed outcomes (Butterfield, 1965). In the case of China’s Cultural Revolution, this framing reduces the movement’s early developments to mere antecedents of later radicalization. Rather than approaching early episodes through the distorting lens of hindsight, this article returns them to their original contexts, foregrounding the ruptures and contingencies that shaped the Cultural Revolution’s unfolding dynamics.

At this moment, several critical questions arise, each warranting further exploration. If, as this article argues, the Cultural Revolution was marked by a significant discontinuity between the first and second halves of 1966, how should we understand the relationship between these distinct episodes? Under what conditions, and through what mechanisms, did the movement’s initial focus on cultural and ideological initiatives give way to a “great political revolution” characterized by mass mobilization, rebellion under the banner of “great democracy,” and direct challenges to party-state institutions? These shifts also call for a reassessment of Mao’s evolving intentions, judgments, and strategies. How did his approach change as the movement unfolded, and how should his role be understood in steering the transition from cultural and ideological purges to full-scale political revolt?

Beyond Mao’s changing intentions, the escalation of what began as a militant campaign against cultural and ideological sectors into an all-encompassing political upheaval was driven by a volatile constellation of developments unfolding both before and after the party plenum in August 1966. While Mao’s role was undeniable, the expansion of the militant cultural movement into a “great political revolution” was neither preconceived nor dictated solely by any single actor. Instead, it took shape through interactions among political actors operating at different levels of the system and proceeded along multiple, partially intersecting pathways.

A critical source of this emerging volatility lay in the ways the early cultural initiative intersected with the institutional legacies and political dynamics carried over from the Socialist Education Movement. In the early summer of 1966, when the Socialist Education Movement’s work-team apparatus was redeployed in schools and universities to implement the militant “cultural revolution,” it transplanted supervisory routines and disciplinary practices that sat uneasily alongside the unrestrained ideological struggle Mao was urging. The result was widespread grassroots conflicts, which both challenged and eroded the party’s bureaucratic authority (Walder, 2009: 28–87). At the same time, the inherited Socialist Education Movement vocabulary of “capitalist roaders” was reactivated in a new political context, as scrutiny turned from putatively bourgeois cultural figures to party officials themselves.

As these tensions accumulated and were set in motion, the shift from a militant cultural movement to a full-scale insurgent assault on the party-state emerged through the entanglement of several processes unfolding in parallel. The cultural offensive interacted with a widening array of ideological and institutional uncertainties, pushing the movement beyond its initial cultural bounds. At the same time, locally rooted student grievances—shaped by longstanding tensions over pedagogy, discipline, and campus governance—were rearticulated in political terms, giving rise to forms of insurgency that exceeded their original scope. Party leaders at various levels further accelerated this drift by recasting Mao’s initial cultural initiative in overtly political terms, thereby expanding the movement’s targets and considerably raising its stakes.^41^ Mao’s responses to these developments were largely reactive and improvisational. Rather than directing a coherent, phased sequence, he grappled with how to interpret and harness events that his own rhetoric had helped unleash. The turn toward a “great political revolution” was thus the conjunctural outcome of cultural militancy, student activism, changing interpretations among party leaders, and Mao’s own reactive interventions—woven together in ways no single political actor fully anticipated or controlled.

It was within this increasingly unstable configuration that Liu Shaoqi’s dramatic fall in early August 1966 emerged as both a contingent outcome of, and a catalyst for, the continuing radicalization of what had begun as a “cultural revolution.” Liu’s deployment of work teams to direct student activism and contain campus unrest exacerbated his existing tensions with Mao. By the Eleventh Plenum, he had been reduced from party stalwart into the embodiment of a “bourgeois line.” In turn, Liu’s fall became both a symbol and a trigger, unleashing insurgent energies that abruptly redefined the Cultural Revolution’s scope and targets. Just months after the purge of Peng Zhen and the Beijing municipal leadership, Liu—state president and Mao’s designated successor—was publicly denounced, signaling that no official could stand above suspicion. Cast as the archetypal “capitalist roader,” Liu became the exemplary reference point for a wave of denunciations and purges. In this sense, Liu’s fall served as a powerful demonstration effect and accelerant, amplifying the movement’s reach and heightening the momentum of escalation already underway (Wu, 2026).

As these dynamics compounded, the boundaries between “cultural” and “political” revolutions blurred beyond recognition. As historians of the French Revolution have long recognized, explaining how a revolution comes into being is analytically distinct from narrating what it becomes.^42^ In this sense, the origins of the Cultural Revolution are one story, too often rewritten in the image of what it later became. To conflate the two is to collapse contingency into inevitability and rupture into design—a reframing that Mao and his inner circle soon sanctioned and retrospectively reclaimed as a “great political revolution.” The result was an unbounded process of escalation. By late 1966, what had begun as a campaign to purge the arts and academia of bourgeois influence had expanded into a wide-ranging and unpredictable political upheaval, engulfing the party-state apparatus as a whole. At a central party conference in late October 1966, Mao acknowledged that the scale of mass radicalism had surprised him, noting that students were causing widespread disruptions. Yet he remained unconcerned, remarking casually, “So be it!” 来了就来了.^43^ His self-assured, almost laissez-faire attitude reflected an extraordinary tolerance for uncertainty and even “chaos,” grounded in the belief that “great disorder under heaven” could ultimately be contained. As subsequent events would demonstrate, however, once social and political contradictions erupted in a concentrated explosion, the resulting loss of control could exceed even Mao’s expectations (Wu, 2014).

In light of these dynamics, this article seeks to deepen understanding of the Cultural Revolution’s origins and early trajectories, without imposing deterministic or teleological closure. Rather than offering a comprehensive account, it highlights interpretive openings that become visible only when the early movement is examined on its own terms. At its core, it argues for treating “cultural revolution” as an analytically distinct category, one that offers a more fruitful historiographical vantage point than conventional narratives allow. By questioning frameworks that flatten rupture and ambiguity, it invites scholars to approach the disparate episodes of the early Cultural Revolution as historically situated processes, whose meanings and political significance were still in formation, unfolding without the coherence or inevitability later narratives often impose.

## Notes

1. For an insightful early critique of this view, see Young, 1986.

2. This article distinguishes analytically between “the Cultural Revolution” (文化大革命, 文革) and “cultural revolution” (文化革命). The former denotes the historically bounded political movement conventionally dated from 1966 to 1976 and defined in both official and scholarly narratives as a unified episode in PRC history. The latter refers more narrowly to the militant cultural initiative launched in the spring and early summer of 1966, prior to the movement’s expansion into a broader political assault on party authority and state institutions. Capitalization throughout reflects this analytical distinction. When contemporaneous documents or speakers are quoted using formal campaign terminology, their phrasing is preserved. Conflating these usages into a single, continuous process risks projecting later political radicalization backward onto an earlier, formative phase whose scope and meaning remained unsettled.

3. On the Socialist Education Movement, see Baum, 1975; MacFarquhar 1997: 334–48.

4. “农村迷信活动的发展影响生产和社会治安” (Superstitious activities in rural areas and their impact on production and public order), 内部参考, December 25, 1962.

5. “迷信活动遍及邯郸工矿区” (Superstitious activities spread throughout the Handan Industrial and Mining District), 内部参考, July 19, 1963.

6. “上海部分青年中的不良倾向” (Unhealthy tendencies among some youth in Shanghai), 内部参考, March 22, 1963; “有些地区宗教对青少年的毒害很深” (In some areas, religion has a profoundly harmful influence on youth), 内部参考, March 8, 1963.

7. “《外国名歌二百首》在津销售情况和问题” (The sales and problems concerning *Two Hundred Famous Foreign Songs* in Tianjin), 内部参考, March 19, 1963.

8. “南昌一些单位舞会成风对青年的腐蚀作用很大” (The spread of dance parties in work units in Nanchang and their corrosive effects on youth), 内部参考, March 29, 1963.

9. “福建不少地区流传坏书刊” (Harmful publications circulating in many areas of Fujian), 内部参考, September 21, 1963; “宁波经过动员促使私人交出黄色书刊几千册” (Ningbo persuaded private individuals to turn in several thousand pornographic publications), 宣教动态, September 2, 1963.

10. “上海部分青年中的不良倾向” (Unhealthy tendencies among some youth in Shanghai), 内部参考, March 22, 1963.

11. “春节谈话纪要” (Record of Spring Festival talks), February 13, 1964; compiled in Unknown, 1968: 94.

12. “接见阿尔巴尼亚妇女代表团和电影工作者的谈话” (Talks with the Albanian women’s delegation and film workers), May 15, 1964; compiled in Unknown, 1968: 113.

13. “中共中央宣传部关于全国文联和各协会整风情况的报告” (Report of the Central Propaganda Department on the rectification of the All-China Federation of Literary and Art Circles and its associations), July 11, 1964; compiled in Documentation Research Office, 1998b: 8–15.

14. “关于文艺问题的讲话” (Remarks on literature and art), January 3, 1964; compiled in People’s Publishing House Reference Materials Office, 1967: 339–51.

15. “刘少奇在华东局和上海市负责干部会议上的讲话” (Speech by Liu Shaoqi at a meeting of responsible cadres of the East China Bureau and the Shanghai Municipal Committee), July 21, 1964; compiled in Song, 2014: n.p.

16. “接见古巴诗人, 作家和艺术家联合会文学部主任比达·罗德里格斯夫妇的谈话” (Conversation with Cuban poet Berta Rodríguez and her husband, head of the Literature Department of the Union of Writers and Artists of Cuba), November 26, 1963; compiled in Unknown, 1968: 67.

17. “接见非洲和拉丁美洲青年学生代表团时的谈话” (Conversation with delegations of African and Latin American youth and students), August 25, 1964; compiled in Unknown, 1968: 168.

18. “接见老挝爱国战线党文工团团长, 副团长和主要成员时的谈话” (Conversation with the head, deputy head, and leading members of the cultural and art troupe of the Lao Patriotic Front Party), September 4, 1964; compiled in Unknown, 1968: 177, 179.

19. “湖南将有计划有步骤地整顿中小学教师队伍” (Hunan will carry out a planned and phased cleanup of the primary and secondary school teaching staff), 内部参考, April 28, 1964.

20. “在社会主义教育运动中小学教师队伍改组问题的情况和意见” (The situation and recommendations concerning the reorganization of the primary and secondary school teaching staff during the Socialist Education Movement), 宣教动态, December 22, 1964.

21. “同参加‘亚非文学交流座谈会’的亚非作家的谈话” (Conversation with Asian and African writers participating in the “Asian-African Literary Symposium”), December 25, 1964; compiled in Unknown, 1968: 203.

22. “接见法国事务部长马尔罗时的谈话” (Conversation with André Malraux, French minister for cultural affairs); compiled in Unknown, 1968: 238–39. For Malraux’s recollection of the exchange, see Malraux, 1968: 378.

23. “毛泽东主席接见阿尔巴尼亚党政代表团谈话记录” (Record of Chairman Mao Zedong’s talks with the Albanian party and government delegation), May 5, 1966; compiled in International Department of the Chinese Communist Party, 1977: 68–69.

24. See Editorial Group of the Biography of Peng Zhen, 2012: 4.468–69; on the formulation and reporting process of the February Outline, see Gong, 2008: 39–64.

25. “中共中央批转文化革命五人小组关于当前学术讨论的汇报提纲” (The Central Committee of the Chinese Communist Party’s approval and transmission of the Cultural Revolution Five-Person Group’s outline report on current academic discussions), February 12, 1966; compiled in Song, 2014: n.p.

26. “林彪同志委托江青同志召开的部队文艺工作座谈会纪要” (Summary of the PLA cultural and artistic work symposium convened by Comrade Jiang Qing on behalf of Comrade Lin Biao), April 10, 1966; compiled in Song, 2014: n.p.

27. “在中央政治局常委扩大会议上的讲话(摘录)” (Excerpts of a speech at the expanded meeting of the Politburo Standing Committee), March 1966; compiled in Unknown, 1968: 257–58.

28. “在政治局扩大会议上的讲话” (Speech at the expanded meeting of the Politburo Standing Committee), March 20, 1966; compiled in Unknown, 1968: 255–57.

29. “与康生等同志谈话纪要” (Minutes of talks with Comrade Kang Sheng and other comrades), March 28–30, 1966; compiled in Unknown, 1968: 258.

30. For a meticulous study of Peng Zhen’s political career prior to the Cultural Revolution, see Zhong, 2024.

31. Lowell Dittmer first suggested that Liu Shaoqi’s downfall may not have been Mao’s original objective in launching the Cultural Revolution but rather an outgrowth of the movement’s unfolding dynamics (Dittmer, 1974). However, the limited sources at the time left Dittmer’s argument tentative, preventing it from gaining scholarly traction. Notably, neither MacFarquhar and Schoenhals’s *Mao’s Last Revolution* nor MacFarquhar’s seminal trilogy, *The Origins of the Cultural Revolution*, engaged with Dittmer’s idea, effectively foreclosing a line of interpretation that points toward a less intentionalist, more contingent reading of Liu’s fate.

32. For a fuller examination of the Liu Shaoqi case, see Wu, 2026.

33. See the recollections of Zhu Yuepeng 朱悦鹏, a *PLA Daily* journalist who participated in drafting the editorial (Zhu, 1994). See also Mu, 1997: 104–105.

34. In this article, Mao invoked the term “cultural revolution” fifteen times, underscoring its conceptual weight. Of the text’s fifteen thematic sections, five directly address issues related to cultural revolution. Two years later, he returned to these concerns in his Yan’an Talks, offering a more elaborate exposition of revolutionary cultural construction.

35. “毛泽东在杭州同胡志明的谈话” (Mao Zedong’s talks with Ho Chi Minh in Hangzhou), June 10, 1966; compiled in International Department of the Chinese Communist Party, 1977: 114–15, 120.

36. “Memorandum of Conversation, between the Delegation of the People’s Republic of China, Led by Comrade Zhou Enlai, and the Leadership of the Party and Government of the People’s Republic of Albania (Excerpts),” June 1966, Wilson Center Digital Archive, https://digitalarchive.wilsoncenter.org/document/117703 (accessed February 8, 2026).

37. For the full text of the Sixteen Articles, see “中国共产党中央委员会关于无产阶级文化大革命的决定” (Decision of the Central Committee of the Chinese Communist Party concerning the Great Proletarian Cultural Revolution), August 8, 1966; compiled in Song, 2014: n.p.

38. At least four articles in the Sixteen Articles directly address issues related to “cultural revolution.” Article 10 calls for the complete elimination of bourgeois intellectuals’ dominance over schools, while Article 11 targets “bourgeois academic authorities” in the scholarly and artistic fields, along with their reactionary viewpoints. Article 12 outlines policies concerning scientific and technical personnel, reflecting broader debates over expertise and ideology. Finally, Article 16 designates Mao’s “On New Democracy” and “Yan’an Talks” as providing the guiding principles for the Cultural Revolution.

39. Mao made this remark during a conversation with an Albanian delegation on February 3, 1967; see also Documentation Research Office, 2013: 6.45.

40. “中共中央批转林彪同志‘八.九’重要讲话” (CCP Central Committee’s approval and circulation of comrade Lin Biao’s important “August 9” speech), October 19, 1967; compiled in Song, 2014: n.p.

41. For an analysis of an early episode in this radicalizing process involving Lin Biao and other senior leaders, see Wu, 2024: 112–46.

42. See Chartier (1991: esp. introduction) on the methodological risks of reading revolutionary rupture backward into its intellectual preconditions.

43. “在中央工作会议上的讲话” (Remarks at the central work conference), October 25, 1966; compiled in Unknown, 1968: 275.
